# Asparagine endopeptidase deficiency mitigates radiation-induced brain injury by suppressing microglia-mediated neuronal senescence

**DOI:** 10.1016/j.isci.2024.109698

**Published:** 2024-04-09

**Authors:** Ouwen Qiu, Jianyi Zhao, Zhonggang Shi, Huan Li, Siyuan Wang, Keman Liao, Minchao Tang, Jieqiong Xie, Xi Huang, Wenrui Zhang, Li Zhou, Xi Yang, Zhiyi Zhou, Lei Xu, Renhua Huang, Yifeng Miao, Yongming Qiu, Yingying Lin

**Affiliations:** 1 Brain Injury Center, Shanghai Institute of Head Trauma, Ren Ji Hospital, Shanghai Jiao Tong University School of Medicine, Shanghai 200127, P.R. China; 2 Department of Radiation Oncology, Ruijin Hospital, Shanghai Jiao Tong University School of Medicine, Shanghai 200025, P.R. China; 3 Department of Hepatobiliary Surgery, Guangxi Medical University Cancer Hospital, Guangxi 530021, P.R. China; 4 Department of Neurology, The Second Affiliated Hospital of Guangxi Medical University, Guangxi 530007, P.R. China; 5 Department of Digestive Oncology, Guangxi Medical University Cancer Hospital, Guangxi 530021, P.R. China; 6 Department of Radiation, Ren Ji Hospital, Shanghai Jiao Tong University School of Medicine, Shanghai 200127, P.R. China; 7 Department of Neurosurgery, Ren Ji Hospital, Shanghai Jiao Tong University School of Medicine, Shanghai 200127, P.R. China

**Keywords:** Molecular biology, Neuroscience, Immunology, Omics, Metabolomics, Transcriptomics

## Abstract

Mounting evidence supports the role of neuroinflammation in radiation-induced brain injury (RIBI), a chronic disease characterized by delayed and progressive neurological impairment. Asparagine endopeptidase (AEP), also known as legumain (LGMN), participates in multiple malignancies and neurodegenerative diseases and may potentially be involved in RIBI. Here, we found AEP expression was substantially elevated in the cortex and hippocampus of wild-type (*Lgmn*^+/+^) mice following whole-brain irradiation. *Lgmn* knockout (*Lgmn*^−/−^) alleviated neurological impairment caused by whole-brain irradiation by suppressing neuronal senescence. Bulk RNA and metabolomic sequencing revealed AEP’s involvement in the antigen processing and presentation pathway and neuroinflammation. This was further confirmed by co-culturing *Lgmn*^+/+^ primary neurons with the conditioned media derived from irradiated *Lgmn*^+/+^ or *Lgmn*^−/−^ primary microglia. Furthermore, esomeprazole inhibited the enzymatic activity of AEP and RIBI. These findings identified AEP as a critical factor of neuroinflammation in RIBI, highlighting the prospect of targeting AEP as a therapeutic approach.

## Highlights

• Knockout of *Lgmn* reduces radiation-induced brain injury
• AEP promotes neuronal senescence via modulating microglia
• Inhibition of AEP's enzymatic activity alleviates radiation-induced brain injury

## Graphical abstract

**Figure:**
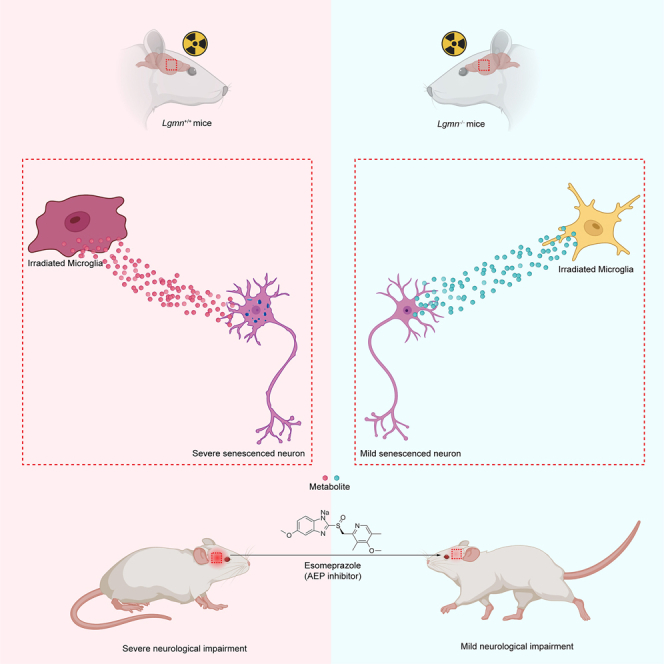

## Introduction

Radiotherapy is a fundamental procedure for treating several types of brain and cerebrovascular diseases.^1^^,^^2^^,^^3^ However, radiotherapy may also cause radiation-induced brain injury (RIBI), which may trigger delayed and progressive neurological complications to some extent.^4^ Many patients experience side effects of RIBI as a result of irradiation of healthy brain tissue adjacent to the nidus, including seizures, cognitive impairment, and so on.^5^^,^^6^^,^^7^ Although this issue has received considerable attention and several up-to-date technical breakthroughs have been achieved and applied in clinical practice, RIBI still poses a challenging conundrum concerning its molecular mechanism, pathophysiology, prevention or effective treatment.^6^^,^^8^ Therefore, it is crucial to address this issue effectively.

Several mechanisms associated with RIBI have been identified, including microglial activation, neuroglial proliferation, and the suppression of neurogenesis.^9^^,^^10^ Moreover, as RIBI causes DNA damage to neurons and other cells in the CNS, cellular senescence may be another mechanism attributed to RIBI.^11^ Cellular senescence is a stress-responsive cell-cycle arrest program that allows proliferating cells to enter a state of permanent cell-cycle arrest when subjected to different stresses.^12^ Key markers of cellular senescence include elevated expression of p21^CIP1^, p16^INK4a^ protein, and the highly enzymatically active β-galactosidase.^13^ Generally, transient cellular senescence recruits immune cells to clear damaged cells, and progenitor cells proliferate and regenerate, achieving tissue remodeling; however, persistent cellular senescence or the inability to clear senescent cells induces chronic inflammation and tissue fibrosis, causing malignant effects.^14^ Additionally, cellular senescence plays an integral role in aging and aging-related diseases, where the marker p16 is elevated in Alzheimer’s (AD) and Parkinson’s disease.^15^^,^^16^^,^^17^ Interestingly, irradiation of the brain also upregulates another pivotal enforcer of senescence, p21, causing DNA damage and driving post-mitotic neuronal senescence.^18^^,^^19^^,^^20^

Furthermore, microglial activation fosters the secretion of different inflammatory factors and chemokines, such as interleukin-1β (IL-1β), tumor necrosis factor-α (TNF-α), C-C motif chemokine ligand 2 (CCL2), and subsequently suppresses neurogenesis, which accounts for another underlying mechanism of RIBI.^21^^,^^22^ Moreover, CCL2 and its receptor, C-C motif chemokine receptor 2 (CCR2), induce sustained activation of microglia following irradiation and trigger the infiltration of CD8^+^ T cells into the brain, releasing cytotoxic factors, such as perforin and granzyme, and causing secondary damage to the brain.^22^^,^^23^^,^^24^ In addition, the conversion of resting astrocytes to A1 astrocytes during RIBI synergizes with activated microglia, secreting pro-inflammatory factors, promoting microvascular injury, and disrupting the integrity of the blood-brain barrier to exacerbate neurological damage.^25^^,^^26^^,^^27^

Regarding therapeutic target of RIBI, asparagine endopeptidase (AEP) is a promising candidate. AEP, also known as legumain (LGMN), functions by specifically cleaving substrates at asparaginyl residues.^28^^,^^29^ Our group has found that AEP participates in the progression and recurrence of gliomas, through cleaving wild type-p53, tropomodulin-3 (Tmod3) and DEAD-box helicase 3 X-linked (DDX3X).^30^^,^^31^^,^^32^ Additionally, AEP is upregulated and activated throughout aging or in degenerative diseases, such as AD.^33^^,^^34^^,^^35^ AEP specifically cleaves tau at residues N255 and N368, inducing tau hyperphosphorylation and aggregation and producing the highly neurotoxic tau 1–368 truncation.^34^ Meanwhile, amyloid-β, which is associated with AD, is generated when AEP specifically proteolytically degrades amyloid-β precursor protein (APP) at N373 and N585 residues.^33^^,^^35^ Moreover, AEP is associated with the regulation of the inflammatory phenotype of macrophages with heterogeneity in different diseases or organs.^36^^,^^37^^,^^38^ However, the interaction between AEP and microglia in RIBI remains vague.

Esomeprazole, a proton pump inhibitor (PPI) used to treat inflammatory diseases of the digestive tract, reduces the enzymatic activity of AEP and suppresses the metastasis of breast cancer mediated by AEP.^39^^,^^40^ Esomeprazole and other PPI drugs can cross the brain-blood barrier, showing their potential to inhibit AEP in the central nervous system (CNS) after intraperitoneal injection.^41^^,^^42^

In our study, *Lgmn*^−/−^ mice were generated and tested in multiple behavioral experiments with *Lgmn*^+/+^ mice to identify the role of AEP in RIBI. To further examine the role of AEP in neuronal senescence, bulk RNA sequencing, metabolomic sequencing, and co-culture of neurons with the conditioned medium derived from irradiated microglia were performed. Furthermore, this study investigated whether RIBI could be alleviated following AEP inhibition via the intraperitoneal injection of esomeprazole.

## Results

### AEP expression was elevated in neurons and microglia in the cortex and hippocampus in RIBI

**Figure 1:**
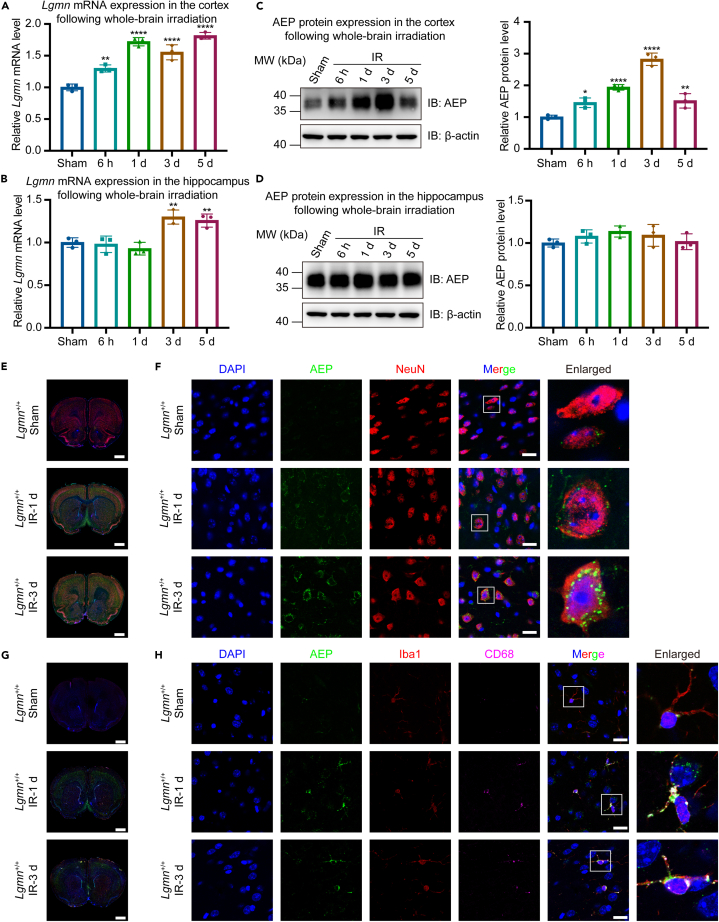
AEP expression was elevated in neurons and microglia in the cortex following whole-brain irradiation (A and B) Relative mRNA levels of *Lgmn* in the cortex (A; F value = 60.60) and hippocampus (B; F value = 14.43) following whole-brain irradiation (*n* = 3 per group). (C and D) Relative protein levels of AEP (left panel) and its quantification (right panel) in the cortex (C; F value = 57.74) and hippocampus (D; F value = 1.24) following whole-brain irradiation (*n* = 3 per group). (E and F) Whole-brain section images (E, scale bar, 1 mm) and localized cortical images (F, scale bar, 20 μm) of immunofluorescence analysis of AEP and NeuN following whole-brain irradiation. (G and H) Whole-brain section images (G, scale bar, 1 mm) and localized cortical images (H, scale bar, 20 μm) of immunofluorescence analysis of AEP, Iba1, and CD68 following whole-brain irradiation. AEP, asparagine endopeptidase; LGMN, legumain; IB, immunoblotting; IR, irradiation; NeuN, neuronal nuclei; Iba1, ionized calcium binding adaptor molecule 1. Data are represented as mean ± SD. One-way ANOVA tests were used in (A–D). ∗*p* < 0.05, ∗∗*p* < 0.01, ∗∗∗∗*p* < 0.0001. See also Figures S1 and S2.

To identify whether AEP participates in pathological processes of RIBI, the cortex and hippocampus were collected and detected for AEP from non-irradiated controls and irradiated BALB/c mice following whole-brain irradiation of a single dose of 10 Gy X-ray. Whole-brain irradiation led to a significant increase in *Lgmn* mRNA expression by 1.30-fold–1.81-fold in the cortex starting at 6 h post-irradiation, whereas the hippocampus only exhibited elevated expression on the 3^rd^ and 5^th^ day following whole-brain irradiation (Figures 1A and 1B). At the protein expression level, immunoblotting confirmed significantly elevated AEP expression >1.46-fold, with a peak of 2.82-fold on the 3^rd^ day post-irradiation in the cortex but not in the hippocampus (Figures 1C and 1D).

Regarding the inconsistency of AEP expression in the cortex and hippocampus following whole-brain irradiation, immunoblotting of AEP was performed 8 weeks following whole-brain irradiation and it showed that AEP expression increased by 1.53-fold and 1.19-fold significantly in the cortex and hippocampus, respectively (Figures S1A and S1B). Considering the significant elevation of AEP in mRNA and protein levels in the cortex following whole-brain irradiation, we focused principally on the role of AEP in the cortex.

Additionally, co-immunofluorescence revealed increased AEP expression in the cortex following whole-brain irradiation. Meanwhile, AEP co-localized with neuronal nuclei (NeuN), ionized calcium binding adaptor molecule 1 (Iba-1), and CD68 but not with Sox9 (Figures 1E–1H, S2A, and S2B), indicating that AEP might play a role in neurons and activated microglia in RIBI.

### Construction and validation of *Lgmn* knockout mice

**Figure 2:**
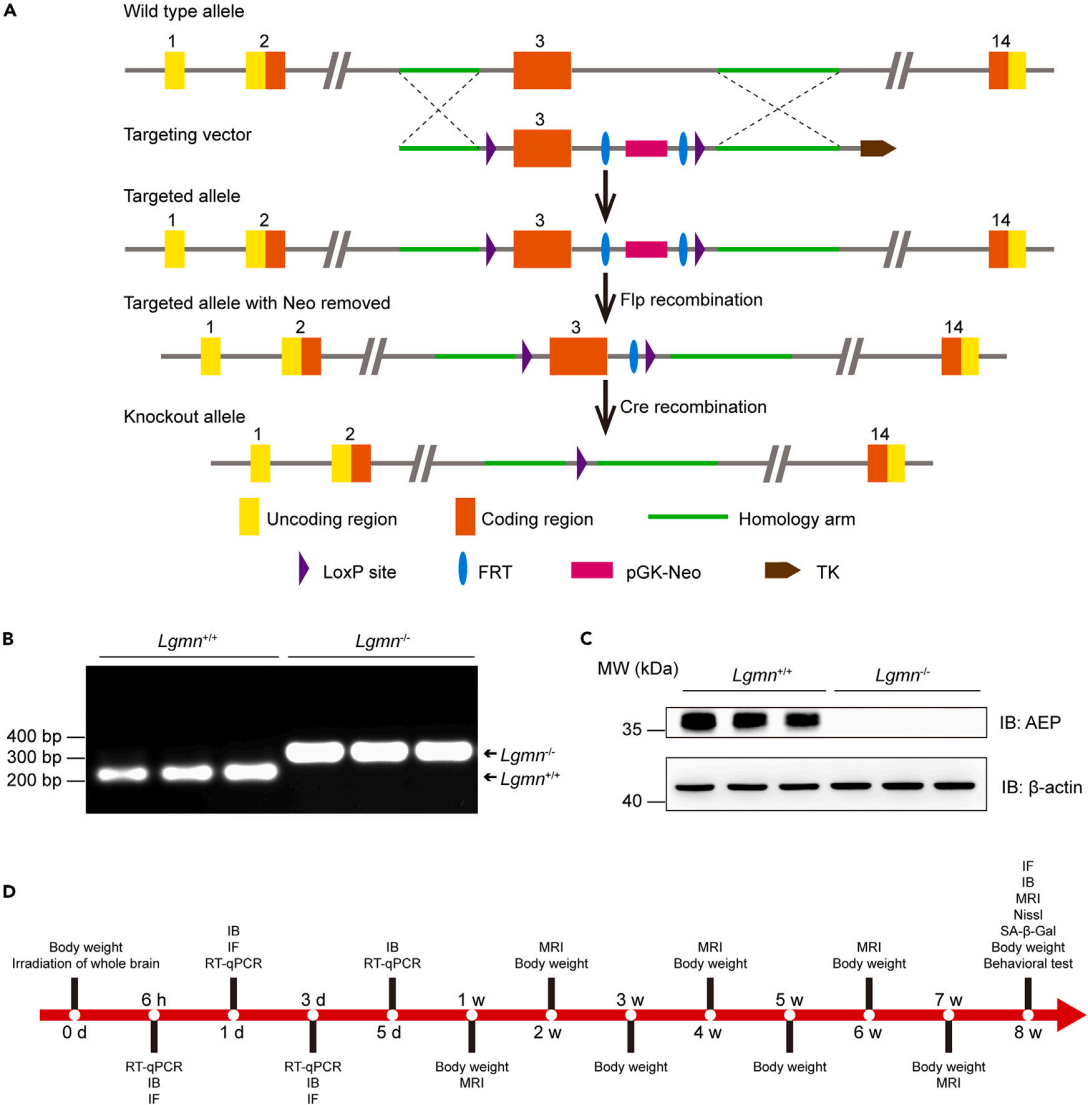
Construction and validation of *Lgmn* knockout mice (A) Schematic diagram of *Lgmn*^−/−^ mice generation. (B) Agarose gel electrophoresis of *Lgmn* from tail of *Lgmn*^+/+^ and *Lgmn*^−/−^ mice (*n* = 3 per group). (C) Immunoblotting analysis of AEP in the cortex of *Lgmn*^+/+^ and *Lgmn*^−/−^ mice (*n* = 3 per group). (D) The experimental design for a part of this study. AEP, asparagine endopeptidase; LGMN, legumain; IB, immunoblotting; IF, immunofluorescence; RT-qPCR, quantitative reverse transcription polymerase chain reaction; SA-β-Gal, senescence-associated β-galactosidase.

To clarify the role of AEP in RIBI, *Lgmn*^−/−^ mice were generated by multiple matings of wild-type (WT) mice with *Lgmn*^*flox*/+^: *Flp*^+^ mice (Figure 2A) and were confirmed by tail genomic DNA validation using 2% agarose gel electrophoresis before animal experiments (Figure 2B). In addition, the expression of AEP in the cortex was detected by immunoblotting, showing a loss of AEP strip at 36 kDa position in *Lgmn*^−/−^ mice in comparison with that in *Lgmn*^+/+^ mice (Figure 2C). After the validation of *Lgmn*^−/−^ mice, *Lgmn*^+/+^, and *Lgmn*^−/−^ mice were whole-brain irradiated. Several molecular, cellular, and behavioral experiments were conducted in *Lgmn*^+/+^-Sham, *Lgmn*^+/+^-irradiated (*Lgmn*^+/+^-IR), *Lgmn*^−/−^-Sham, and *Lgmn*^−/−^-irradiated (*Lgmn*^−/−^-IR) mice at different time points to explore the role of AEP in RIBI (Figure 2D).

### *Lgmn* knockout reduced the damage to the brain in RIBI

To discover the intuitive damage to the brain following whole-brain irradiation, MRI scans were applied to *Lgmn*^+/+^-Sham, *Lgmn*^+/+^-IR and *Lgmn*^−/−^-IR mice at various intervals following whole-brain irradiation. The injury to the brain could not be clearly observed in the 1^st^ or 4^th^ week following whole-brain irradiation (Figures S3A and S3B). Six weeks following whole-brain irradiation, MRI scan images displayed higher signal intensity in the cortex of *Lgmn*^+/+^-IR mice by 1.29-fold than that of *Lgmn*^+/+^-Sham mice, indicating cerebral edema associated with RIBI (Figures S3C and S3D). This effect decreased after *Lgmn* depletion, demonstrating its potentially effective role in mitigating RIBI. This tendency became more significant 8 weeks following whole-brain irradiation (Figures S3E and S3F).

### *Lgmn* knockout alleviated the neurological impairment in RIBI

Whole-brain irradiation can impair the neurological functions in mice. Therefore, several cortex-related and hippocampus-related behavioral tests were performed in *Lgmn*^+/+^-Sham, *Lgmn*^+/+^-IR, *Lgmn*^−/−^-Sham, and *Lgmn*^−/−^-IR mice.

**Figure 3:**
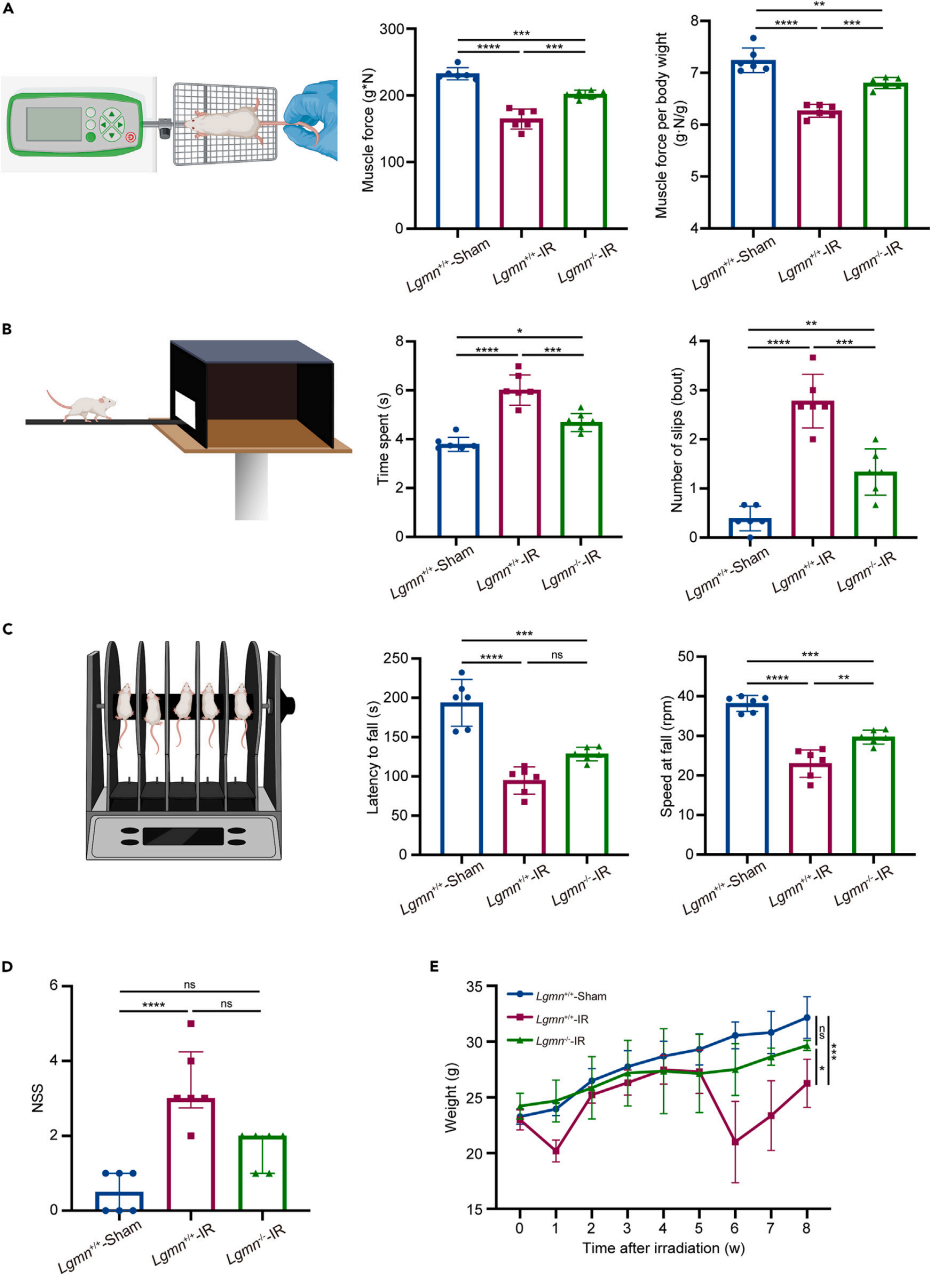
The neurological impairment following whole-brain irradiation was reduced by *Lgmn* knockout (A) Muscle force tests (middle and right panel; F value of genetic type = 35.81 and 31.58 in the middle and right panel respectively; F value of irradiation = 119.60 and 104.30 in the middle and right panel, respectively) and the schematic diagram (left panel) of *Lgmn*^+/+^-Sham, *Lgmn*^+/+^-IR and *Lgmn*^−/−^-IR mice (*n* = 6 per group). (B) Balance beam tests (middle and right panel; F value of genetic type = 26.48 and 32.29 in the middle and right panel, respectively; F value of irradiation = 74.12 and 88.33 in the middle and right panel, respectively) and the schematic diagram (left panel) of *Lgmn*^+/+^-Sham, *Lgmn*^+/+^-IR and *Lgmn*^−/−^-IR mice (*n* = 6 per group). The middle panel is the time spent traveling the distance of 80 cm of the beam, and the right panel is the number of slips that happened during the travel. (C) Rotarod tests (middle and right panel; F value of genetic type = 8.07 and 21.35 in the middle and right panel, respectively; F value of irradiation = 69.50 and 108.90 in the middle and right panel, respectively) and the schematic diagram (left panel) of *Lgmn*^+/+^-Sham, *Lgmn*^+/+^-IR and *Lgmn*^−/−^-IR mice (*n* = 6 per group). The middle panel is the latency of mice falling from the rod, and the right panel is the speed of the rod when mice fell. (D) Neurological severity score tests of *Lgmn*^+/+^-Sham, *Lgmn*^+/+^-IR and *Lgmn*^−/−^-IR mice (*n* = 6 per group). (E) Body weight of *Lgmn*^+/+^-Sham, *Lgmn*^+/+^-IR and *Lgmn*^−/−^-IR mice (*n* = 6 per group; F value of genetic type = 12.47; F value of irradiation = 37.41). LGMN, legumain; IR, irradiation; NSS, neurological severity score. Data are represented as median ± IQR in (D), whereas the other data are represented as mean ± SD. two-way ANOVA tests were used in (A–C and E), and Kruskal-Wallis tests were used in (D). ∗*p* < 0.05, ∗∗*p* < 0.01, ∗∗∗*p* < 0.001, ∗∗∗∗*p* < 0.0001, ns = not significant. See also Figures S3–S5 and Video S1.

First, muscle force, balance beam, rotarod and neurological severity score tests were conducted with the record of body weight. As the *Lgmn* knockout did not significantly influence the results of these behavioral experiments and body weight between *Lgmn*^+/+^-Sham and *Lgmn*^−/−^-Sham mice (Figure S4A–S4E), the same experiments were performed in *Lgmn*^+/+^-IR and *Lgmn*^−/−^-IR mice 8 weeks following irradiation, along with *Lgmn*^+/+^-Sham mice. Muscle force experiments revealed reduced muscle force by approximately 29.19% following irradiation; this reduction was alleviated by *Lgmn* knockout and was still significant after normalization of body weight (Figure 3A). Moreover, in the balance beam test, *Lgmn*^+/+^-IR mice spent an average of 2.25 more s passing an 80 cm beam than *Lgmn*^+/+^-Sham mice with more times of slips, whereas *Lgmn*^−/−^-IR mice displayed better performance than that of *Lgmn*^+/+^-IR mice (Figure 3B). In the rotarod test, *Lgmn*^+/+^-IR mice more easily fell off the rotarod at a slower speed and maintained an average of 98.78 s fewer than *Lgmn*^+/+^-Sham mice, whereas *Lgmn*^−/−^-IR mice maintained a tendency of more time than *Lgmn*^+/+^-IR mice could (Figure 3C and Video S1). The neurological severity score (NSS) test showed the most severe neurological injury following whole-brain irradiation in *Lgmn*^+/+^-IR mice among the three groups, whereas *Lgmn*^−/−^-IR mice showed a tendency of mitigated neurological injury in comparison with *Lgmn*^+/+^-IR mice (Figure 3D). Interestingly, *Lgmn*^+/+^-IR mice presented a significant loss of body weight of nearly 18.35% at 8 weeks following whole-brain irradiation, whereas depletion of *Lgmn* alleviated this weight loss by nearly 57.80% (Figure 3E).

Video S1. Video of balance beam tests in *Lgmn*^+/+^-Sham, *Lgmn*^+/+^-IR and *Lgmn*^-/-^-IR mice, related to Figure 3

Moreover, open field, novel object recognition and Barnes maze tests were conducted. Open field test showed that whole-brain irradiation reduced the total distance moved in the field, the distance moved in the center and the time spent in the center, whereas *Lgmn*^−/−^ mice presented more activities in the center in comparison with *Lgmn*^+/+^ mice (Figure S5A). However, there was no significant difference in recognition and discrimination index found in the novel recognition test between *Lgmn*^+/+^ and *Lgmn*^−/−^ groups (Figure S5B). In Barnes maze test, whole-brain irradiated mice spent more time and distance to reach the target hole with less time in the target quadrant in comparison to sham mice. *Lgmn* knockout mice spent significantly less distance to the target hole, but they spent only a tendency of less time to reach the target hole and more time in the target quadrant without significant difference (Figures S5C and S5D).

Thus, these behavioral results suggest that *Lgmn* knockout alleviates the neurological impairment following whole-brain irradiation.

### *Lgmn* knockout decreased neuronal senescence in RIBI

The senescence pathway is regulated in pathological processes associated with irradiation in the CNS^18^^,^^19^^,^^20^; therefore, whether AEP was involved in irradiation-induced neuronal senescence was examined and experiments detecting the expression of p21, p16, and senescence-associated beta-galactosidase (SA-β-Gal) staining were conducted.

**Figure 4:**
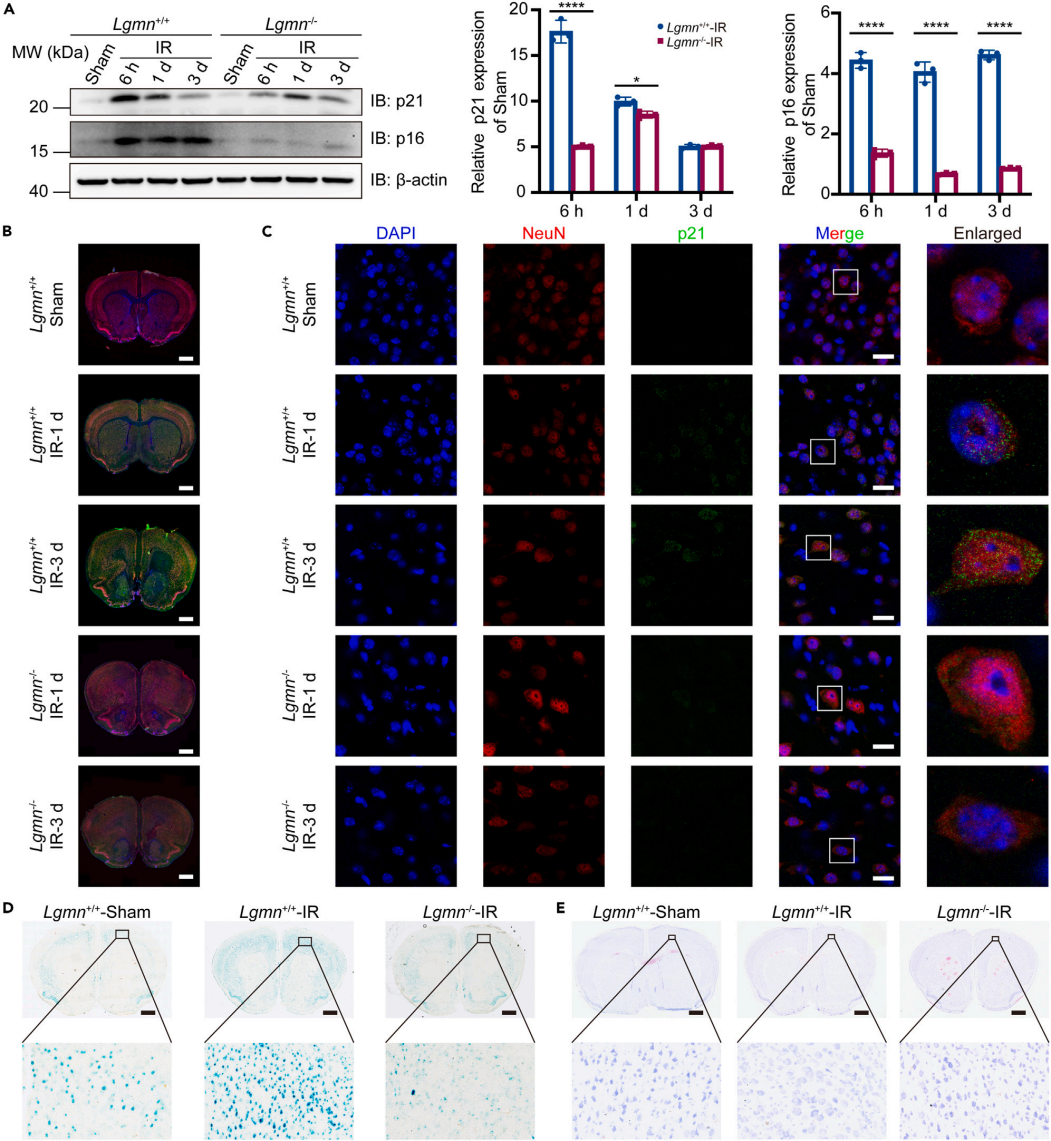
Neuronal senescence following whole-brain irradiation was reduced by *Lgmn* knockout (A) Immunoblotting (left panel) and the quantification (middle and right panel) of p21 and p16 from the cortex following whole-brain irradiation (*n* = 3 per group). (B and C) Whole-brain section images (B, scale bar, 1 mm) and localized cortical images (C, scale bar, 20 μm) of immunofluorescence analysis of p21 and NeuN following whole-brain irradiation. (D) SA-β-Gal staining of frozen brain sections from *Lgmn*^+/+^-Sham, *Lgmn*^+/+^-IR and *Lgmn*^−/−^-IR mice 8 weeks following whole-brain irradiation (scale bar, 1 mm). (E) Nissl staining of frozen brain sections from *Lgmn*^+/+^-Sham, *Lgmn*^+/+^-IR and *Lgmn*^−/−^-IR mice 8 weeks following whole-brain irradiation (scale bar, 1 mm). LGMN, legumain; IR, irradiation; SA-β-Gal, senescence-associated β-galactosidase. Data are represented as mean ± SD. Student’s *t* tests were used in (A). ∗*p* < 0.05, ∗∗∗∗*p* < 0.0001.

Following whole-brain irradiation, the expression of p21 and p16 was significantly elevated in the cortex of *Lgmn*^+/+^-IR mice, and was rescued by *Lgmn* depletion (Figure 4A). Immunofluorescence revealed higher expression of p21 in *Lgmn*^+/+^-IR mice following whole-brain irradiation than in *Lgmn*^−/−^-IR mice and colocalization of p21 and NeuN (Figures 4B and 4C). In addition, compared to the cortex of *Lgmn*^−/−^-IR mice, the cortex of *Lgmn*^+/+^-IR mice were more positively stained in SA-β-Gal staining 8 weeks following irradiation (Figure 4D). Collectively, these results suggest that following whole-brain irradiation, *Lgmn* knockout decreases neuronal senescence. Moreover, Nissl staining revealed that Nissl bodies were less reduced in the cortex of *Lgmn*^−/−^-IR mice following whole-brain irradiation than in that of *Lgmn*^+/+^-IR mice, indicating a protective effect of the *Lgmn* knockout against RIBI (Figure 4E).

### AEP regulated RIBI via the antigen processing and presentation pathway

**Figure 5:**
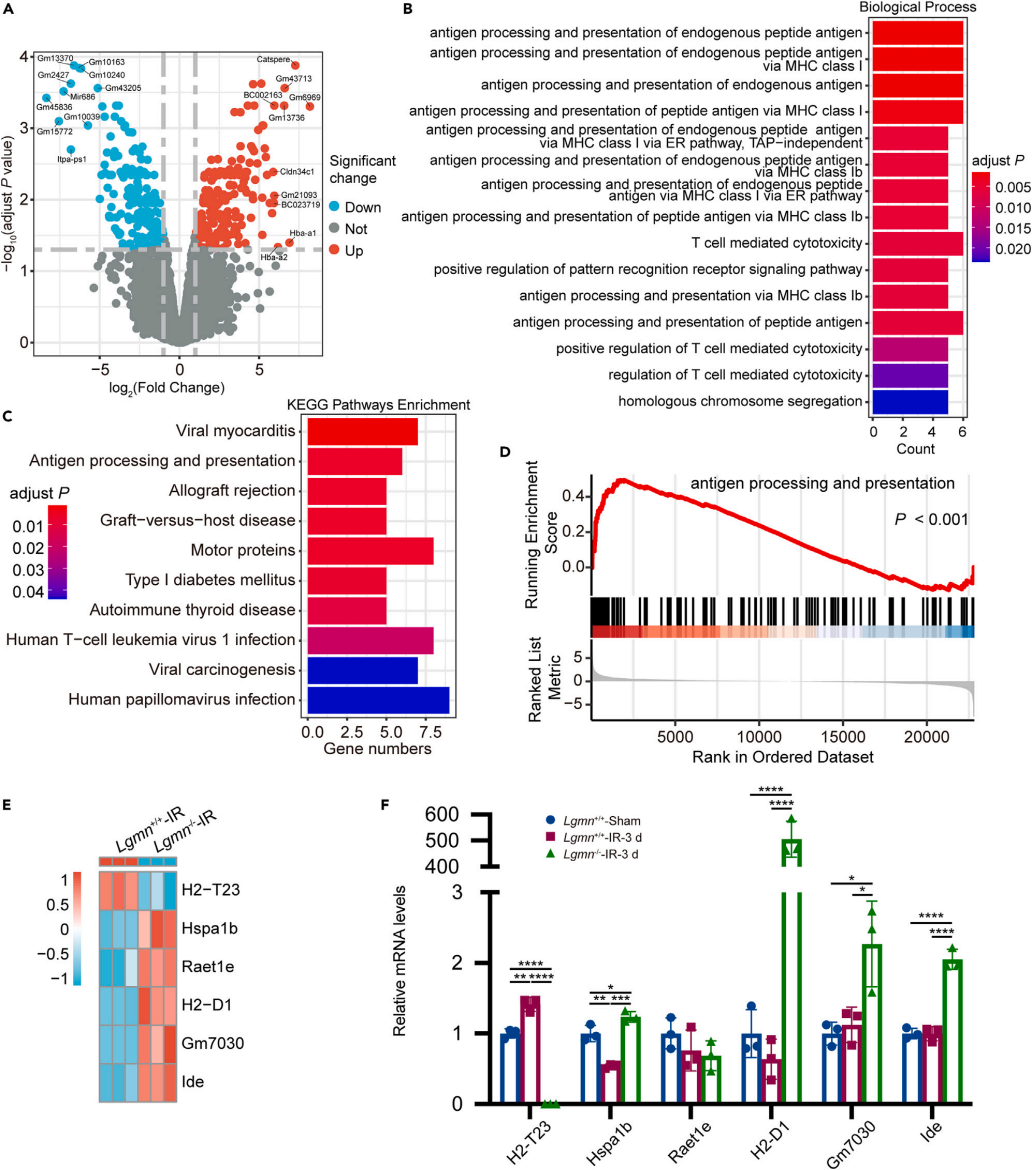
AEP modulated radiation-induced brain injury via the antigen processing and presentation pathway (A) Volcano plot displays the DEGs from the cortex between *Lgmn*^+/+^-IR and *Lgmn*^−/−^-IR mice 3 days following whole-brain irradiation. (B) GO biological process analysis of the significant DEGs from the cortex between *Lgmn*^+/+^-IR and *Lgmn*^−/−^-IR mice 3 days following whole-brain irradiation. (C) KEGG pathway analysis of the significant DEGs from the cortex between *Lgmn*^+/+^-IR and *Lgmn*^−/−^-IR mice 3 days following whole-brain irradiation. (D) GSEA analysis of the antigen processing and presentation pathway. (E) Heatmap of DEGs clustered in the antigen processing and presentation pathway. (F) Validation of the mRNA levels of the genes enriched in the antigen processing and presentation pathway in the cortex from *Lgmn*^+/+^-Sham, *Lgmn*^+/+^-IR-3d and *Lgmn*^−/−^-IR-3d mice (*n* = 3 per group; F value of genetic type = 623.20, 112.30, 0.14, 239.80, 12.96 and 137.40, respectively; F value of irradiation = 53.56, 49.29, 1.47, <0.01, 0.15 and <0.01, respectively). AEP, asparagine endopeptidase; LGMN, legumain; IR, irradiation; DEGs, differentially expressed genes; GO, gene ontology; KEGG, Kyoto Encyclopedia of Genes and Genomes; GSEA, gene set enrichment analysis. Data are represented as mean ± SD. two-way ANOVA tests were used in (F). ∗*p* < 0.05, ∗∗*p* < 0.01, ∗∗∗*p* < 0.001, ∗∗∗∗*p* < 0.0001. See also Figure S6.

To determine the pathway by which AEP affects neuronal senescence following whole-brain irradiation, the cortex of *Lgmn*^+/+^-IR and *Lgmn*^−/−^-IR mice were extracted 3 days following whole-brain irradiation and subjected to bulk RNA sequencing. Among the differentially expressed genes (DEGs) between *Lgmn*^+/+^-IR and *Lgmn*^−/−^-IR mice, the volcano plot displays 211 upregulated and 224 downregulated genes (Figure 5A). The heatmap displays the top 50 up- and downregulated genes (Figure S6). Subsequently, DEGs were subjected to functional enrichment analysis to explore potential signaling pathways. Gene ontology (GO) biological process enrichment and Kyoto Encyclopedia of Genes and Genomes (KEGG) pathway enrichment revealed several significant signaling pathways, including antigen processing and presentation, T cell-mediated cytotoxicity, homologous chromosome segregation, and allograft rejection pathways (Figures 5B and 5C). In addition, the molecular mechanism mediated by AEP in RIBI was investigated using gene set enrichment analysis (GSEA), and the antigen processing and presentation pathway was identified (Figure 5D). The DEGs clustered in this pathway consisted of *H2-T23*, *Hspa1b*, *Raet1e*, *H2-D1*, *Gm7030*, and *Ide* (Figure 5E) and were verified by RT-qPCR (Figure 5F).

### AEP modulated neuronal senescence by interacting with microglia following irradiation

**Figure 6:**
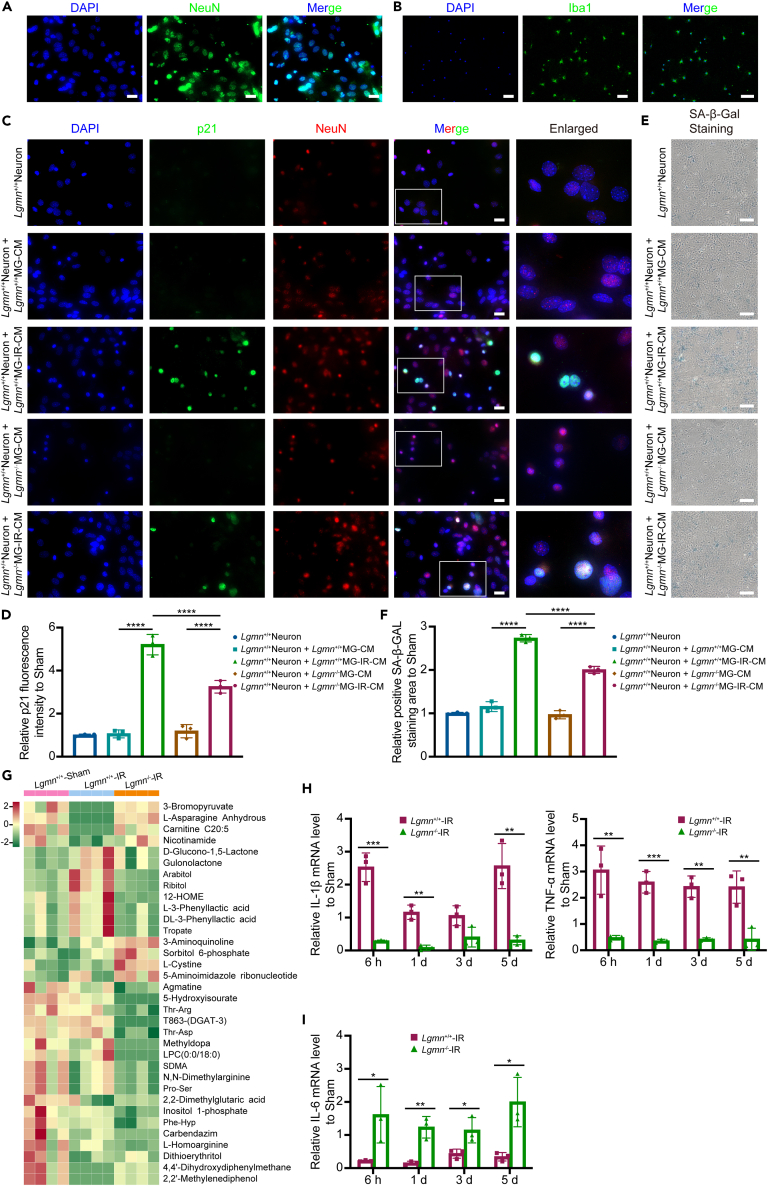
AEP modulated neuronal senescence by interacting with microglia following irradiation (A) Validation of primary neurons by immunofluorescence analysis of NeuN (scale bar, 20 μm). (B) Validation of primary microglia by immunofluorescence analysis of Iba1 (scale bar, 100 μm). (C and D) Immunofluorescence analysis (C, scale bar, 20 μm) and the quantification (D and F value of genetic type = 5.48; F value of irradiation = 62.88) of p21 and NeuN in primary neurons cocultured with or without the conditioned medium from microglia. (E and F) SA-β-Gal staining (E, scale bar, 100 μm) and the quantification (F; F value of genetic type = 19.49; F value of irradiation = 158.00) of primary neurons cocultured with or without the conditioned medium from microglia. (G) Heatmap of the significant differential metabolites in the culture medium of primary *Lgmn*^+/+^-Sham, *Lgmn*^+/+^-IR and *Lgmn*^−/−^-IR microglia. (H and I) Relative mRNA levels of pro-inflammatory factors IL-1β, TNF-α (H) and anti-inflammatory factor IL-6 (I) in the cortex of *Lgmn*^+/+^-IR and *Lgmn*^−/−^-IR mice (*n* = 3 per group). AEP, asparagine endopeptidase; LGMN, legumain; MG, microglia; CM, conditioned medium; IR, irradiation; SA-β-Gal, senescence-associated β-galactosidase. Data are represented as mean ± SD. two-way ANOVA tests were used in (D and F), and Student’s *t* tests were used in (H and I). ∗*p* < 0.05, ∗∗*p* < 0.01, ∗∗∗*p* < 0.001, ∗∗∗∗*p* < 0.0001.

As AEP was verified to be involved in the antigen processing and presentation pathway, we wonder whether microglia, the main cells responsible for antigen processing and presentation in the CNS, would interact with neuronal senescence following irradiation. First, primary neurons and primary microglia were extracted from neonatal mouse cortex and validated by immunofluorescence staining with 4',6-diamidino-2-phenylindole (DAPI), anti-NeuN or anti-Iba1, the specific antibodies presented by neurons or microglia, respectively. The purity of both cells was >95% (Figures 6A and 6B). *Lgmn*^+/+^ and *Lgmn*^−/−^ microglia were irradiated and their culture medium supernatant was used to culture *Lgmn*^+/+^ neurons. The expression of p21 was significantly elevated in neurons one day after co-culturing with the medium conditioned by irradiated *Lgmn*^+/+^ microglia and was rescued by *Lgmn* knockout (Figures 6C and 6D). Neuronal senescence was also confirmed by SA-β-Gal staining 3 days after co-culture, where knockout of *Lgmn* inhibited the elevated level of neuronal senescence (Figures 6E and 6F).

To identify the microglial metabolites influencing neuronal senescence following irradiation, metabolomic sequencing was conducted in the conditioned media from *Lgmn*^+/+^-Sham, *Lgmn*^+/+^-IR and *Lgmn*^−/−^-IR microglia, and 34 significantly different metabolites were discovered among these three groups. Four metabolites (3-bromopyruvate, L-asparagine anhydrous, carnitine C20:5, and nicotinamide) were first decreased following irradiation in the conditioned medium of *Lgmn*^+/+^-IR microglia than those in *Lgmn*^+/+^-Sham microglia, and then increased by knockout of *Lgmn* in *Lgmn*^−/−^-IR microglia, whereas another eight metabolites (D-glucono-1,5-lactone, gulonolactone, arabitol, ribitol, 12-HOME, L-3-phenyllactic acid, DL-3-phenyllactic acid, and tropate) were the opposite (Figure 6G). Most of these metabolites are involved in the regulation of inflammation in the immune microenvironment. Considering that AEP may participate in the regulation of inflammation in a variety of diseases,^36^^,^^37^^,^^38^ RT-qPCR was performed in the cortex of *Lgmn*^+/+^-Sham, *Lgmn*^+/+^-IR, and *Lgmn*^−/−^-IR mice, revealing that whole-brain irradiation led to an increased expression of pro-inflammatory factors *Il-1β* and *Tnf-α* and a decreased expression of anti-inflammatory factor *Il-6*, which were notably reversed by knockout of *Lgmn* (Figures 6H and 6I).

### Esomeprazole mitigated RIBI by inhibiting AEP’s enzymatic activity

**Figure 7:**
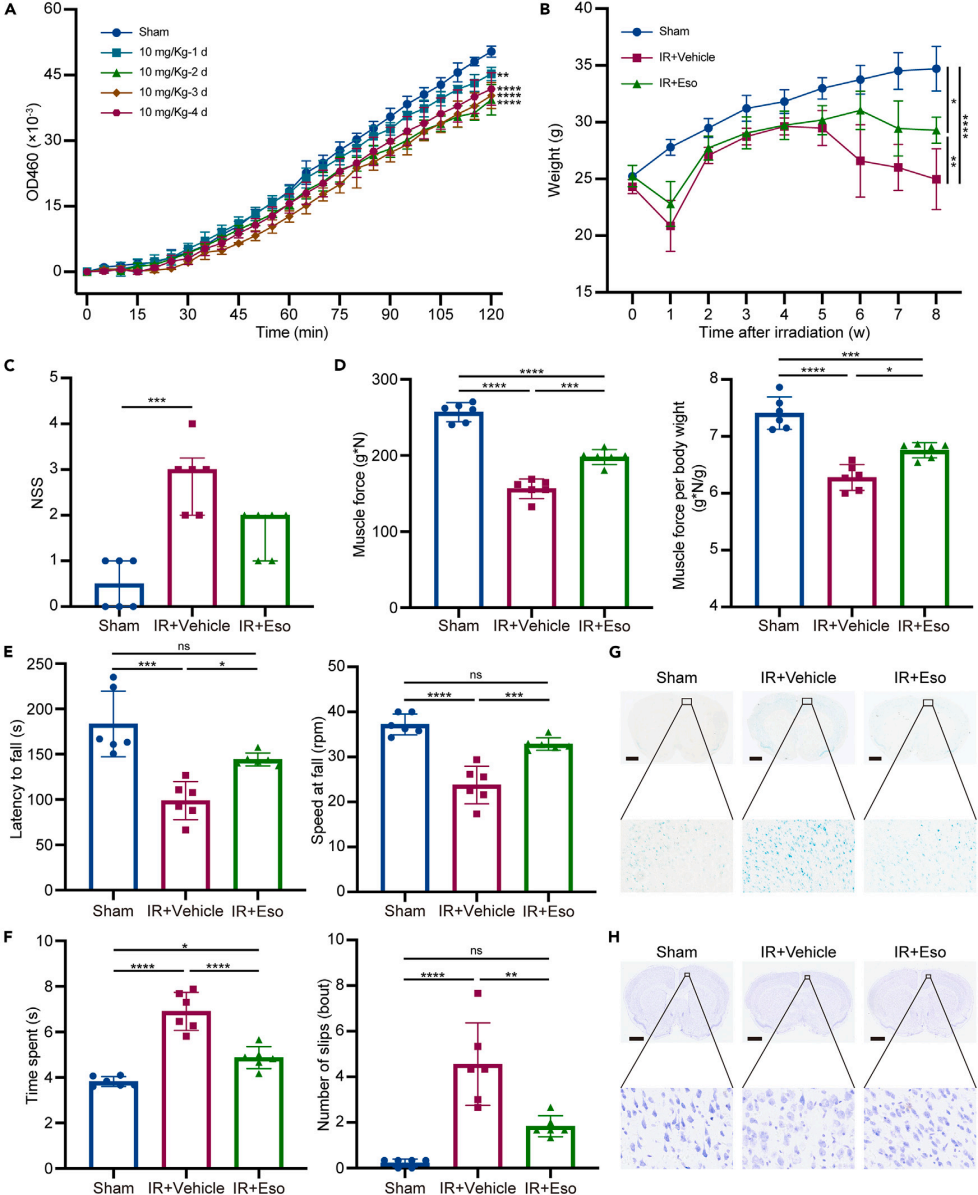
Esomeprazole mitigated radiation-induced brain injury by inhibiting AEP’s enzymatic activity (A) AEP’s enzymatic activity in the cortex of WT mice with or without intraperitoneal injection of 10 mg/kg esomeprazole (*n* = 3 per group; F value = 47.63). (B) Body weight of Sham, IR + Vehicle and IR + Eso mice (*n* = 6 per group; F value of esomeprazole = 12.47; F value of irradiation = 37.41). (C) Neurological severity score tests of Sham, IR + Vehicle and IR + Eso mice (*n* = 6 per group). (D) Muscle force tests of Sham, IR + Vehicle and IR + Eso mice (*n* = 6 per group; F value of esomeprazole = 37.44 and 13.74 in the left and right panel, respectively; F value of irradiation = 218.10 and 76.80 in the left and right panel, respectively). (E) Rotarod tests of Sham, IR + Vehicle and IR + Eso mice. The left panel is the latency of mice falling from the rod, and the right panel is the speed of the rod when mice fell (*n* = 6 per group; F value of esomeprazole = 10.32 and 29.85 in the left and right panel, respectively; F value of irradiation = 35.75 and 65.38 in the left and right panel respectively). (F) Balance beam tests of Sham, IR + Vehicle and IR + Eso mice. The left panel is the time spent traveling the distance of 80 cm of the beam, and the right panel is the number of slips that happened during the travel (*n* = 6 per group; F value of esomeprazole = 37.89 and 18.98 in the left and right panel respectively; F value of irradiation = 87.14 and 48.08 in the left and right panel, respectively). (G) SA-β-Gal staining of frozen brain sections from Sham, IR + Vehicle and IR + Eso mice 8 weeks following whole-brain irradiation (scale bar, 1 mm). (H) Nissl staining of frozen brain sections from Sham, IR + Vehicle and IR + Eso mice 8 weeks following whole-brain irradiation (scale bar, 1 mm). AEP, asparagine endopeptidase; IR, irradiation; Eso, esomeprazole; NSS, neurological severity score; SA-β-Gal, senescence-associated β-galactosidase. Data are represented as median ± IQR in (C), whereas the other data are represented as mean ± SD. One-way ANOVA tests were used in (A), two-way ANOVA tstes were used in (B, D–F), and Kruskal-Wallis tests were used in (C). ∗*p* < 0.05, ∗∗*p* < 0.01, ∗∗∗*p* < 0.001, ∗∗∗∗*p* < 0.0001, ns = not significant. See also Figure S7.

AEP acts as a protein hydrolase, and its enzymatic activity can be inhibited by PPI drugs, which suppress breast cancer metastasis.^39^^,^^40^ To determine whether esomeprazole is capable of inhibiting the enzymatic activity of AEP in the CNS, intraperitoneal injections of 10 and 20 mg/kg esomeprazole were administered daily to mice, and we found that 10 mg/kg esomeprazole exhibited better potential (Figures 7A and S7). Therefore, 10 mg/kg esomeprazole was daily administrated to *Lgmn*^+/+^-IR mice (IR + Eso), while saline vehicle of the same dosage was administered to *Lgmn*^+/+^-IR (IR + Vehicle) and *Lgmn*^+/+^-Sham mice (Sham) daily, respectively, for 8 weeks. Furthermore, several behavioral experiments were conducted to explore the effectiveness of esomeprazole application in relieving RIBI.

Results of behavioral experiments between IR + Vehicle and Sham mice were similar to those between *Lgmn*^+/+^-IR mice and *Lgmn*^+/+^-Sham mice (Figures 7B–7F). Like the depletion of *Lgmn*, the inhibition of AEP’s enzymatic activity by esomeprazole in IR + Eso mice alleviated whole-brain irradiation-induced body weight loss and elevated neurological severity scores (NSS) when compared with IR + Vehicle mice (Figures 7B and 7C). Additionally, esomeprazole administration relieved the irradiation-induced muscle force loss by 41.43% and 40.00% when normalized to body weight (Figure 7D). Moreover, IR + Eso mice sustained 45.50 s more than IR + Vehicle mice did on the rotarod and 38.32% quicker speed of rotarod before falling (Figure 7E). Furthermore, IR + Eso mice spent 2.03 s less than IR + Vehicle mice did in passing the 80 cm balance beam, with 59.86% fewer slips (Figure 7F). At the cellular level, esomeprazole inhibited irradiation-induced neuronal senescence and loss of Nissl bodies (Figures 7G and 7H). Collectively, these findings suggest that inhibiting the enzymatic activity of AEP via the intraperitoneal injection of esomeprazole might alleviate RIBI.

## Discussion

To clarify the effect of AEP on RIBI, WT mice were exposed to whole-brain irradiation and detected aberrantly increased mRNA and protein expression levels of AEP, as well as neurological deficits relevant to RIBI. Co-culturing of neurons with the conditioned medium from irradiated microglia confirmed the role of AEP in mediating the antigen processing and presentation pathway of microglia. Metabolomic sequencing revealed the role of AEP in neuroinflammation in RIBI. Blockade of AEP by transgenic depletion or esomeprazole administration effectively rescued irradiation-induced neuronal senescence and mitigated the associated neurological impairment.

RIBI observed on MRI, including necrotic lesions and edema, was reported to appear at 6 weeks and become apparent 8 weeks post-irradiation in mice in a previous study.^24^ However, in our study, only signal intensity differences and not organic lesions were detected. This variability may be explained by the localized gamma-ray irradiation at 50 Gy used in that study, which was five times stronger than ours, causing severer damage that was easier to identify using MRI.^24^ This study chose dose of X-rays in whole-brain irradiation to be 10 Gy, considering the idea that the peritumoral normal brain tissue may receive a small dose of radiation during radiotherapy in patients with brain tumors, which is consistent with previous literature reports.^23^ Moreover, mice exposed to whole-brain irradiation at 10 Gy in this study did not exhibit MRI-detectable lesions at 8 weeks post-irradiation or even at 12 weeks (data not shown). We speculate that a time of more than 12 weeks cannot detect whole-brain irradiation-induced brain lesions, as mice may not be viable until they develop MRI-visible lesions owing to the excessive damage caused by whole-brain irradiation.

Regarding the mechanisms associated with RIBI, microglia-related inflammation of the CNS plays an important role in RIBI.^22^^,^^23^^,^^43^^,^^44^^,^^45^ Although microglial activation initially contributes to the phagocytosis of dead cells, sustained microglial activation would activate the chronic inflammatory state of the brain.^46^^,^^47^ Increased levels of inflammatory factors and polarization of microglia toward the pro-inflammatory (M1) phenotype after brain irradiation may be associated with neuronal damage and cognitive decline.^21^^,^^48^^,^^49^^,^^50^ This study investigated the activation of microglia following irradiation; this activation promotes neuronal senescence and impairs cortical function in mice.

AEP plays an important role in the polarization of pro-inflammatory (M1) and anti-inflammatory (M2) phenotypes in macrophages/microglia. AEP is overexpressed by anti-inflammatory (M2) macrophages and plays an anti-fibrotic role in obstructive nephritis, whereas it exhibits a pro-inflammatory (M1) phenotype in carotid atherosclerosis and worsens the disease.^36^^,^^37^ Although different studies hold different views on AEP-mediated macrophage polarization outside the CNS, the current studies suggest that AEP primarily mediates microglial polarization toward pro-inflammatory (M1) phenotype and promotes inflammation in the CNS. Particularly in AD, the knockout of *Lgmn* in mice alleviates neuroinflammation and AD pathologies, indicating the pro-inflammatory effect of AEP in the brain.^38^ Similar to AD, irradiation of the brain upregulated the expression of AEP and promoted the neuroinflammatory effect of microglia, whereas *Lgmn* knockdown attenuated neuroinflammation and mitigated neurological deficits.

Bulk RNA sequencing revealed that *Lgmn* knockdown resulted in changes to the expression profiles of several genes in the irradiated cortex, of which the *H2-T23* was downregulated in *Lgmn*^−/−^-IR mice, whereas the *Hspa1b*, *Raet1e*, *H2-D1*, *Gm70770*, and *Ide* were upregulated in *Lgmn*^−/−^-IR mice. It is reported that *H2-T23* is a nonclassical major histocompatibility complex Ib (MHC Ib) class molecule in mice and is involved in the regulation of immune responses through interactions with inhibitory receptors of CD8^+^ T cells and natural killer (NK) cells.^51^ Moreover, *H2-D1* is a major component of the MHC I class of molecules, which are expressed by neurons and microglia and are modulated during aging in mice, and are also associated with the regulation of pro-inflammatory genes.^52^ Meta-analysis of aging data from human brains also suggests that the expression of MHC I is age-related. The induction of MHC I signaling with age may lead to altered synaptic regulation and impaired synaptic plasticity. The results of bulk RNA sequencing in this study suggest that AEP can modulate the inflammatory environment within the CNS through the MHC I pathway and affect neuronal senescence, and thus mediate RIBI.

Furthermore, metabolites are crucial to disease progression. For instance, 3-bromopyruvate is neuroprotective in aging-related disorders because of its antioxidant potential at the mitochondrial level and autophagy regulation.^53^ Most tumor cells depend highly on aerobic glycolysis rather than mitochondrial oxidative phosphorylation for energy. Interestingly, 3-bromopyruvate, a small-molecule alkylating agent that is a potential inhibitor of glycolysis, targets the process of glycolysis without inhibiting the oxidative phosphorylation of mitochondria in tumor cells, and thus inhibits tumor cells without affecting the function of normal cells.^54^ Moreover, eicosapentaenoic acid (carnitine C20:5) reduces inflammation and oxidative stress in AD.^55^ Nicotinamide administration can induce elevated NAD^+^ levels, which can alleviate inflammation, improve aging-related functional deficits, and combat neurodegenerative diseases.^56^^,^^57^ Furthermore, L-asparagine deprivation inhibits protein synthesis and metabolism.^58^ Metabolomic sequencing in this study revealed that 3-bromopyruvate, carnitine C20:5, nicotinamide, and anhydrous L-asparagine decreased following whole-brain irradiation but were rescued by *Lgmn* depletion. Therefore, it is highly likely that whole-brain irradiation induces metabolic changes in microglia, reinforcing RIBI, whereas depletion of *Lgmn* rescues this progression.

The role of AEP varies. AEP is associated with myocardial infarction, malignancies, and neurodegenerative diseases.^30^^,^^31^^,^^32^^,^^33^^,^^59^ Moreover, in ischemia and seizure, AEP triggers DNA damage by cleaving DNase.^60^ In addition, *Lgmn* knockout reduced the level of depression-like and anxiety-like behavior, and augmented the spatial cognition.^61^ Here, we discovered the protective role of *Lgmn* knockout in RIBI and the related neurological impairments. Although AEP did not increase immediately in the hippocampus following whole-brain irradiation significantly, its protein expression was elevated 8 weeks later, and it partially alleviated whole-brain irradiation-induced hippocampus injury.

The glioblastoma’s tumor microenvironment causes senescence of the adjoining normal brain tissue after radiotherapy, and creates a senescence-associated secretory phenotype and promotes tumor recurrence in turn.^20^ Interestingly, p21 depletion abrogated irradiation-induced senescence and inhibited tumor progression and recurrence.^20^ Given the pro-tumor role of AEP in glioma via the cleavage of wild type-p53, Tmod3, and DDX3X, AEP also serves as a potential target for treating glioma.^30^^,^^31^^,^^32^ Therefore, in conjunction with our experimental results, the inhibition of AEP reduced tumor progression and decreased senescence-induced tumor recurrence in the CNS after radiotherapy. This dual antitumor effect makes AEP a potential therapeutic target for the treatment of glioma.

AEP exerts its enzymatic activity mainly in its cleaved form and specifically cleaves peptide bonds at asparaginyl residue.^28^^,^^29^ PPI medications function as direct covalent inhibitors of AEP through disulfide bonds with the sulfhydryl (SH) group of the protease active site,^40^^,^^41^ the effect of which in the CNS was confirmed by our results. Employing esomeprazole at 10 mg/kg minimized RIBI by inhibiting the enzymatic activity of AEP and could be considered a potential therapy for preventing RIBI. AEP has been reported to specifically cleave SET, an inhibitor of DNase, and thus induces DNA damage during seizures and cerebral ischemia, which may represent a possible mechanism by which AEP regulates RIBI.^60^ However, the specific AEP substrate that mediates RIBI requires further investigation.

In summary, our investigation revealed that AEP plays an important role in RIBI via mediating neuronal senescence by targeting microglia. Targeting AEP by pharmaceutical application of the PPI esomeprazole is promising for alleviating RIBI.

### Limitations of the study

AEP exerts its function by specific cleavage of substrates at asparaginyl residues. In this study, inhibition of the enzymatic activity of AEP alleviated RIBI, demonstrating its enzymatic function in the regulation of RIBI. However, the specific substrate that might be cleaved by AEP to regulate RIBI has not yet been identified and requires further exploration. In addition, all animal experiments were performed in male mice, where may exist some bias, and further parallel animal experiments in female mice are required.

## STAR★Methods

### Key resources table

**Table:** 

REAGENT or RESOURCE	SOURCE	IDENTIFIER
**Antibodies**		

Sheep anti-mouse legumain polyclonal antibody, unconjugated	R and D Systems	Cat# AF2058; RRID:AB_2234536
Mouse monoclonal anti-beta actin (HRP conjugated)	Abways	Cat# AB2001; RRID:AB_3076723
Rabbit polyclonal anti-p21	Proteintech	Cat# 28248-1-AP; RRID:AB_2881097
Rabbit monoclonal anti-p16INK4a	Abcepta	Cat# AP11690b; RRID:AB_10818914
Goat polyclonal anti-rabbit IgG H&L (HRP conjugated)	Abcam	Cat# ab6721; RRID:AB_955447
Rabbit polyclonal anti-sheep IgG H&L (HRP conjugated)	Abcam	Cat# ab6747; RRID:AB_955453
Mouse monoclonal anti-NeuN	Cell Signaling Technology	Cat# 94403; RRID:AB_2904530
Rabbit monoclonal anti-SOX9	Huabio	Cat# ET1611-56; RRID:AB_2924312
Rabbit monoclonal anti-Iba1	Cell Signaling Technology	Cat# 17198; RRID:AB_2820254
Rat monoclonal anti-CD68	Bio-Rad	Cat# MCA1957; RRID:AB_322219
Goat polyclonal anti-Iba1	FUJIFILM Wako Shibayagi	Cat# 011–27991; RRID:AB_2935833
Donkey polyclonal anti-sheep IgG H&L (Alexa Fluor® 488)	Abcam	Cat# ab150177; RRID:AB_2801320
Donkey polyclonal anti-mouse IgG H&L (Alexa Fluor® 594)	Abcam	Cat# ab150108; RRID:AB_2732073
Donkey polyclonal anti-goat IgG H&L (Alexa Fluor® 594)	Abcam	Cat# ab150132; RRID:AB_2810222
Donkey polyclonal anti-rabbit IgG H&L (Alexa Fluor® 488)	Abcam	Cat# ab150073; RRID:AB_2636877
Donkey polyclonal anti-rabbit IgG H&L (Alexa Fluor® 594)	Abcam	Cat# ab150076; RRID:AB_2782993
Donkey polyclonal anti-rat IgG H&L (Alexa Fluor® 647)	Abcam	Cat# ab150155; RRID:AB_2813835

**Chemicals, peptides, and recombinant proteins**		

TRIzol	Invitrogen	Cat# 15596026
RIPA buffer	Solarbio Life Sciences	Cat# R0010
EDTA-free protease inhibitor cocktail	Roche	Cat# 4693132001
PVDF membrane	Millipore	Cat# IPVH00010
Donkey serum	Solarbio Life Sciences	Cat# SL050
DAPI staining solution	Beyotime Biotech. Inc.	Cat# C1006
Nissl staining solution	Beyotime Biotech. Inc.	Cat# C0117
Neutral balsam mounting medium	Sangon Biotech	Cat# E675007
Anti-fade mounting medium	Sangon Biotech	Cat# E675011
Papain	Sigma-Aldrich	Cat# P4762
DNase I	Roche	Cat# 11284932001
Neurobasal-A medium	Gibco	Cat# 10888022
B27 supplement without vitamin A	Gibco	Cat# 12587010
Fetal bovine serum	Gibco	Cat# 10100147
GlutaMAX	Gibco	Cat# 35050079
Penicillin-streptomycin	Gibco	Cat# 15140122
Poly-D-lysine	Sigma-Aldrich	Cat# P6407
Trypsin	Gibco	Cat# 15050065
Dulbecco’s modified Eagle medium	Gibco	Cat# 11965092
Z-Ala-Ala-Asn-AMC	MCE	Cat# HY-136626

**Critical commercial assays**		

HiScript II 1st strand cDNA synthesis kit (+gDNA wiper)	Vazyme BioTech	Cat# R212-02
ChamQ SYBR qPCR master mix	Vazyme BioTech	Cat# Q341-02
Mouse tail direct PCR kit	Share-Bio	Cat# SB-201
Senescence β-galactosidase staining kit	Beyotime	Cat# C0602

**Deposited data**		

Raw and analyzed data of bulk RNA sequencing	This paper	GEO: GSE246261
Raw and analyzed data of metabolomic sequencing	This paper	MetaboLights: MTBLS9647

**Experimental models: Cell lines**		

Mouse primary cortical neuron	This paper	N/A
Mouse primary cortical microglia	This paper	N/A

**Experimental models: Organisms/strains**		

Mouse: *Lgmn*^+/+^: BALB/c	This paper	N/A
Mouse: *Lgmn*^−/−^: BALB/c	This paper	N/A
*Lgmn*^*flox*/+^: *Flp*^+^: BALB/c mouse	Shanghai Model Organisms Center, Inc.	N/A
BALB/c mouse	Shanghai JieSiJie Laboratory Animal Co., Ltd.	N/A

**Oligonucleotides**		

Primers used in this study, see Tables S1 and S2	This paper	N/A

**Software and algorithms**		

Image Lab 6.1.0	Bio-Rad Laboratoried, Inc.	https://www.bio-rad.com/en-cn/product/image-lab-software
RadiAnt DICOM Viewer 2023.1	Medixant	https://www.radiantviewer.com/
VisuTrack XR-VT	Shanghai XinRuan Information Technology Co.	https://www.shxinruan.com/
GraphPad Prism 10.0.3	GraphPad Software	https://www.graphpad.com/
RStudio 2023.06.01 + 524	Posit Software, PBC	https://www.rstudio.com/categories/rstudio-ide/
R 4.3.1	The R Foundation for Statistical Computing	https://www.r-project.org

### Resource availability

#### Lead contact

Further information and requests for resources and reagents should be directed to and will be fulfilled by the lead contact, Yingying Lin (yylin@sibs.ac.cn).

#### Materials availability

Mouse lines generated in this study are available upon request from the lead contact, Yingying Lin (yylin@sibs.ac.cn).

#### Data and code availability

• Bulk RNA-seq data and metabolomic sequencing have been deposited at GEO and MetaboLights respectively, and are publicly available as of the date of publication. Accession numbers are listed in the key resources table. Original western blot images and microscopy data reported in this paper are available from the lead contact upon request.
• No original code has been generated in this study.
• Any additional information required to reanalyze the data reported in this paper is available from the lead contact upon request.

### Experimental model and study participant details

#### Animals and ethics statement

WT BALB/c mice were purchased from Shanghai JieSiJie Laboratory Animal Co., Ltd. *Lgmn*^*flo*x/+^: *Flp*^+^ mice were generated by the Shanghai Model Organisms Center, Inc. *Lgmn*^+/+^ BALB/c mice (male, 6–8 weeks old) and *Lgmn*^−/−^ mice (male, 6–8 weeks old) were used in this study. All mice were housed in an SPF environment with a 12-h light-dark cycle, in addition to sufficient regular laboratory mouse diet and water. Same-sex littermates were randomly assigned to different experimental groups. This study was approved by the Ethics Committee of Ren Ji Hospital (RA-2022-185). No effort was spared to refine the procedure to reduce the number and suffering of mice used in the experiment.

#### Primary cell cultures

Primary cortical neurons and microglia were isolated from the cortex of neonatal male *Lgmn*^+/+^ or *Lgmn*^−/−^ mice under 2 days of age. Primary neurons and microglia were cultured in a humidified 37°C and 5% CO_2_ incubator. The purity of primary neurons and microglia was detected by immunofluorescence analysis using anti-NeuN and anti-Iba1, respectively.

### Method details

#### Genetic editing of mice

Briefly, ES cell targeting vector was constructed using the Infusion method, which contains 3.0 kb 5′ homologous arm, 0.7 kb flox region, PGK-Neo-polyA, 3.0 kb 3′ homologous arm and MC1-TK-polyA negative screening marker. The vector was linearized and electro-transfected into ES cells. A positive clone with the correct homologous recombination was obtained by long-fragment PCR identification. The positive ES cell clone was amplified and injected into blastocysts of BALB/c mice to generate chimeric mice. A high percentage of chimeric mice were mated with Flp mice to obtain five positive F1 generations of de-Neo heterozygous (*Lgmn*^*flox*/+^: *Flp*^+^) mice.

After acquiring *Lgmn*^*flox*/+^: *Flp*^+^ mice, *Lgmn*^*flox*/+^ mice were obtained by mating *Lgmn*^*flox*/+^: *Flp*^+^ mice and WT mice, and then mating with Cre mice to generate *Lgmn*^*flox*/+^: *Cre*^+^ mice. Finally, *Lgmn*^*flox*/+^ mice were mated with *Lgmn*^*flox*/+^: *Cre*^+^ mice to generate *Lgmn*^*flox/flox*^: *Cre*^+^ (*Lgmn*^−/−^) mice.

#### Culture of primary neurons and microglia

Neonatal male mice under 2 days of age were euthanized, and their cortices were obtained under sterile conditions on ice.

For culture of primary neurons, the cortex was dissected into small pieces with sharp scissors followed by 1 mg/mL papain with 0.5 μg/mL DNase Ⅰ for 30 min at 37°C, stopped by and suspended in the Neurobasal-A medium containing 2% B27, 5% FBS, 0.25× GlutaMAX and 1% penicillin-streptomycin. Cells were then plated on 24-well plates with slide coated with 0.1 mg/mL PDL at the density of 1 × 10^5^ cells/cm^2^ in a humidified 37°C and 5% CO_2_ incubator. The medium was changed to the Neurobasal-A medium containing 1% penicillin-streptomycin the next day, and it was changed by half every 3 days. The purity of primary neurons was determined by immunofluorescence analysis with anti-NeuN.

Primary microglial culture was performed based on previous protocols.^62^^,^^63^ Briefly, the cortex was dissected into small pieces with sharp scissors followed by 0.125% Trypsin with 0.25 μg/mL DNase Ⅰ for 15 min at 37°C, stopped by and suspended in the DMEM medium containing 10% FBS and 1% penicillin-streptomycin. Cells were then plated on cell culture flasks coated with 0.01 mg/mL PDL at the density of 5 × 10^4^ cells/cm^2^ in a humidified 37°C and 5% CO_2_ incubator. The medium was changed the following day to remove cellular debris. After astrocytes achieved confluence and formed the layer supporting the growth of microglia, microglia were mechanically isolated by shaking the flask at 200 rpm for 2 h at 37°C. The collected microglia were plated and purified through differential adherence by replacing the culture medium to remove loosely adhering cells 30 min after plating in an incubator. The medium was changed the next day and every 5 days thereafter. The purity of primary microglia was determined by immunofluorescence analysis with anti-Iba1.

For the co-culture experiment, primary *Lgmn*^+/+^ and *Lgmn*^−/−^ microglia were first subjected to a single 4 Gy dose of X-rays. 24 h later, the culture medium supernatant of the irradiated microglia was used to culture primary *Lgmn*^+/+^ neurons.

#### Tail genomic DNA validation

Genomic DNA was obtained from mouse tails using a mouse tail direct PCR kit, according to the manufacturer’s instructions. *Lgmn* was amplified by PCR, separated by 2% agarose gel electrophoresis, and visualized using a ChemiDoc Imaging System (1708370, Bio-Rad, Richmond, California). The primer sequences are listed in Table S1.

#### Irradiation

The whole brain of each mouse was exposed to a single 10 Gy dose of radiation (6MV X-rays, 600 MU/min) using a medical linear accelerator (Elekta Synergy, Stockholm, Sweden) with 0° and 180° fields and a field size of 3 × 40 cm.

Primary cortical microglia were exposed to a single 4 Gy dose of radiation (6MV X-rays, 600 MU/min) using a medical linear accelerator (Elekta Synergy, Stockholm, Sweden) with 180° fields and a field size of 40 cm × 40 cm.

#### RNA extraction and transcript RT-qPCR analysis

Total RNA was extracted from the cortex and hippocampus using TRIzol Reagent (Invitrogen, Carlsbad, California), and cDNA was synthesized using a cDNA synthesis kit according to the manufacturer’s instructions. Gene expression analysis was conducted on a LightCycler 480 System (Roche, Basel, Switzerland) using the qPCR master mix reagent according to the manufacturer’s instructions. The primer sequences are listed in Table S2.

#### Immunoblotting analysis

Briefly, the cortex and hippocampus of mice were lysed in RIPA buffer containing 1/10 protease inhibitor cocktail tablet dissolved in solution, 1 mM PMSF, 1 mM Na_3_VO_4_, and 10 mM NaF, and the proteins were separated by a 10% SDS-PAGE gel via electrophoresis. Next, the proteins were electro-transferred onto PVDF membranes and blocked with 5% non-fat milk in TBST for 1 h at room temperature. Subsequently, the membranes were incubated with the indicated primary antibody at 4°C overnight. The next day, the membranes were washed three times with TBST for 5 min each and incubated with the indicated secondary antibody conjugated with HRP for 1 h at room temperature. Finally, signals were detected with a chemiluminescent HRP substrate and analyzed using Image Lab software. Each test was repeated three times.

#### Immunofluorescence analysis

Mice brains were fixed in 4% paraformaldehyde and sliced into 25 μm sections using a frozen sectioning machine (CM1950, Leica Microsystems, Wetzlar, Germany). The brain sections were permeabilized using 1% PBST (1 mL Triton X-100 in 100 mL PBS), followed by blocking with 0.3% PBST containing 10% donkey serum for 4 h at room temperature. Then, they were incubated with the indicated primary antibodies at 4°C overnight, followed by incubation of secondary antibodies at room temperature for 3 h after extensive washing with 0.3% PBST. Next, brain sections were counterstained with DAPI at room temperature for 15 min. After extensive washing, the coverslips were mounted using an anti-fade mounting medium. Signals were detected using confocal fluorescence microscopy.

Immunofluorescence analysis of cells was performed on cells adhered to slides Cell slides were gently rinsed three times with PBS, followed by fixation by using 4% PFA for 15 min at room temperature. Then cell slides were permeabilized using 0.5% PBST, followed by blocking with 0.3% PBST containing 10% donkey serum for 1 h at room temperature. Afterward, they were incubated with the indicated primary antibodies at 4°C overnight, followed by incubation of secondary antibodies at room temperature for 1 h after extensive washing with 0.3% PBST. Next, cell slides were counterstained with DAPI at room temperature for 5 min. After extensive washing, cell slides were mounted using an anti-fade mounting medium. Signals were detected using confocal fluorescence microscopy.

#### Brain MRI

Damage to the brains of mice following whole-brain irradiation was detected by 7.0 T or 3.0 T MRI scan with contrast. The signal intensity of the MRI image was quantified by RadiAnt DICOM Viewer.

#### Bulk RNA sequencing and functional enrichment analysis

Three cortical samples from *Lgmn*^+/+^-IR and *Lgmn*^−/−^-IR mice 3 days following whole-brain irradiation were quickly frozen using liquid nitrogen. Total RNA from each sample was extracted using a TRIzol kit and used to generate a cDNA library. The cDNA was sequenced by using a DNBSEQ-T7 sequencer (Sangon Biotech Co., Ltd., Shanghai, China). The raw reads were filtered using Trimmomatic (version 0.36) and mapped to the reference genome using HISAT2 (version 2.0). The gene expression values of the transcripts were computed using StringTie (version 1.3.3b). Significantly DEGs were analyzed using the limma R package in R studio, and genes with adjusted *p* value <0.05 and |log_2_(fold change)| > 1 were identified as DEGs. Functional enrichment for GO, KEGG, and GSEA were analyzed using the clusterProfiler R package with an adjusted *p* value <0.05. These results were visualized using the ggplot 2, pheatmap, and enrichplot R packages.

#### Metabolomic sequencing

Four samples of the cell culture supernatant from primary *Lgmn*^+/+^-IR and *Lgmn*^−/−^-IR microglia, 24 h after a single dose of 4 Gy irradiation, along with the cell supernatant from primary *Lgmn*^+/+^-Sham microglia, were quickly frozen using liquid nitrogen. All samples were first freeze-dried and reconstituted with a solution (Methanol: Water = 4:1, v/v) containing an internal standard, and then concentrated for LC-MS analysis by ExionLC AD and QTRAP 6500 (Metware Biotech Co., Ltd., Wuhan, China). To analyze the differential metabolites, the data were log_2_-transformed and analyzed using the MetaboAnalystR software package (Version 1.0.1) in R studio to obtain the OPLS-DA data. Variable importance in the projection (VIP) values were extracted from these data, and metabolites with VIP >1 and ANOVA *p* value <0.05 were defined as differential metabolites. The expression levels of differential metabolites were Z-score-normalized and presented as a heatmap. The identified differential metabolites were annotated using the KEGG Compound Database (https://www.kegg.jp/kegg/compound/) and mapped to the KEGG Pathway Database (https://www.kegg.jp/kegg/pathway.html). The mapped pathways with obvious regulatory roles were analyzed for metabolite enrichment.

#### Enzymatic activity assay of AEP

To measure the enzymatic activity of AEP, a specific fluorescent substrate, Z-Ala-Ala-Asn-AMC was used and cleaved by AEP to generate fluorescent AMC, based on a previous study with modification.^34^ Briefly, cortex homogenates (300 μg) were incubated with 20 μM Z-Ala-Ala-Asn-AMC in the 200 μL assay buffer (60 mM Na_2_HPO_4_, 20 mM citric acid, 1 mM EDTA, 0.1% CHAPS, and 1 mM DTT, pH 6.0). Results were quantified by measuring fluorescent intensity at 460 nm at 37°C every 5 min for 2 h in the kinetic mode.

#### Behavioral experiments

All mice were fondled for at least 10 min daily for 3 days before behavioral experiments to minimize the stress response. All behavioral experiments were performed at 10 a.m. in the same test order for each mouse. Each equipment was cleaned with 70% ethanol and dried after each test. The body weight of each mouse was measured and recorded every 7 days. Behavioral experiments were performed in a double-blind situation without knowledge of the numbering of mice or groupings to reduce bias due to subjective errors in the experimental data.

#### Muscle force test

The mouse was placed on the grip net and gently and evenly pulled back into the tail until it released the grip net. The maximum grip strength for each mouse was recorded. This experiment was repeated three times. Body weights were measured simultaneously.

#### Balance beam test

Mice were placed on one side of a 0.6 cm-wide wooden beam with a bright bulb above the mice. Mice were trained daily to crawl the beam to the opposite side and enter the dark safe box to escape light 3 days before the official experiment. During the official experiment, the time spent traveling 80 cm from the beam and the number of slips that occurred were documented and analyzed. The experiment was repeated three times for each mouse at an interval of at least 15 s.

#### Rotarod test

Mice were placed on the rod of a rotarod test apparatus. The rod was accelerated uniformly from 4 to 40 rpm in 3 min and maintained at 40 rpm for another 2 min. The latency time taken for the mice to fall from the rotarod and the speed of the rotarod when the mice fell were recorded during a 5-min period. The experiment was repeated three times for each mouse at an interval of at least 15 min.

#### Neurological severity score test

To test the neurological severity of brain injury following irradiation, a modified neurological severity score (NSS) test was applied to each mouse 8 weeks post-irradiation.^64^ The NSS consists of 10 items used to evaluate the neurological performance of each mouse, including motor ability, balance, and alertness (Table S3). Failure to complete each task item adds one point, up to a maximum of 10 points.

#### Open field test

An open field (50 cm L × 50 cm W × 40 cm H) was equally divided into 16 parts, with four middle parts considered the central area. Mice were placed in the center and allowed to move freely for 5 min, while their locomotor activities were automatically recorded by a visual-tracking system. The distance traveled and the time spent in the central and the whole area were analyzed.

#### Novel object recognition test

Novel object recognition tests were conducted based on previous studies with modifications.^65^^,^^66^^,^^67^ Briefly, a mouse was first placed in an open field (50 cm L × 50 cm W × 40 cm H) and allowed to move freely for 5 min for habituation. Then, the mouse was kept in its home cage for 24 h. Afterward, during the familiarization session, two identical objects were placed in the open field, each 8 cm away from the walls. The mouse was placed in the field, with its head positioned opposite the objects, and was allowed to move freely for 5 min. After 1-h rest in its home cage after this familiarization session, one of the objects was changed to a novel object with a different shape. The mouse was placed in the field and allowed to move freely for 5 min, and their activities were automatically recorded by a visual-tracking system in this test session. When the mouse pointed its nose toward an object with a distance less than 2 cm, it was defined as exploring the object. The time of exploring the familiar (a) and novel object (b) was analyzed. The recognition index is defined as a/(a+b) and the discrimination index is defined as (b-a)/(a+b).

#### Barnes maze test

Barnes maze tests were conducted based on a previous study with minor modifications.^68^ The maze was made of a circular 90 cm diameter black PVC slab, with 20 holes of 5 cm diameter equally spaced in the perimeter of the maze, 2 cm from the edge. An escape cage was placed under one of these 20 holes. After each experiment, the equipment was cleaned with 70% ethanol, and the PVC slab was rotated clockwise to avoid odor cues, whereas the escape cage remained in the same position.

During the habituation (day 1) and training (day 2 and 3) phases, a mouse was first placed in the center of the maze within a PVC pipe for 15 s. The mouse was then given 3 min to explore the maze. If the mouse managed to enter the target hole within 3 min, the mouse was allowed to rest in the escape cage for another 2 min. Otherwise, the mouse was gently guided toward it and given another 2 min to rest in the escape cage. Each mouse received training once on day 1, three times on day 2, and twice on day 3. During the probe (day 4) phase, the mouse was placed in the center of the maze within a PVC pipe for 15 s, and was allowed to explore the maze for 3 min. The probe phase ended after 3 min or when the mouse reached the target hole. During the probe phase, the trace of the path, time spent per quadrant, latency to enter the target hole and distance traveled were recorded by a visual-tracking system.

#### SA-β-Gal staining

The senescence of neurons and frozen brain sections was assessed by an SA-β-Gal staining kit according to the manufacturer’s manual. Briefly, frozen brain sections or neurons adhered on slides were rinsed three times with PBS, followed by fixation for 15 min at room temperature. After another three times of rinse with PBS, they were incubated with the SA-β-Gal staining solution at 37°C overnight. Finally, the samples were sealed and photographed under a bright field for subsequent analysis.

#### Nissl staining

Frozen brain sections were stained using Nissl staining solution according to the manufacturer’s instructions. Briefly, frozen brain sections were fixed in 4% PFA for 10 min, followed by two rinses with distilled water for 2 min each. Subsequently, they were stained with Nissl staining solution for ∼8 min at 37°C. Afterward, they were rinsed twice with distilled water twice and dehydrated using 95% ethanol twice for 2 min each. Finally, the sections were cleared in xylene twice for 5 min each and sealed with neutral balsam mounting medium.

### Quantification and statistical analysis

Quantitative data were first tested for normality. Mean ± standard deviation was used to describe data that conformed to a normal distribution, otherwise, median ± inter-quartile range was used to describe data that did not conform to a normal distribution. When comparing the means of the two groups of quantitative data, Student’s t test was used in data that were from a normally distributed population, otherwise, the nonparametric Mann-Whitney U-test was used. Multiple group comparisons were conducted using one-way or two-way analysis of variance (ANOVA) when data were normally distributed with homogeneity, and post-hoc tests were conducted using Dunnett’s and Tukey’s test respectively; otherwise, Kruskal-Wallis test was performed with Dunn’s test. All statistical analyses were performed using GraphPad Prism with α of 0.05 bilaterally unless otherwise stated. *p* < 0.05 was considered significant.
