# Supplementary material for: Asparagine endopeptidase deficiency mitigates radiation-induced brain injury by suppressing microglia-mediated neuronal senescence
Source: iScience. 2024 Apr 9;27(5):109698. doi: 10.1016/j.isci.2024.109698 (PMC11035374; doi:10.1016/j.isci.2024.109698)
Supplement: Document S1. Figures S1–S7 and Tables S1–S3 [file mmc1.pdf]

Supplemental Information

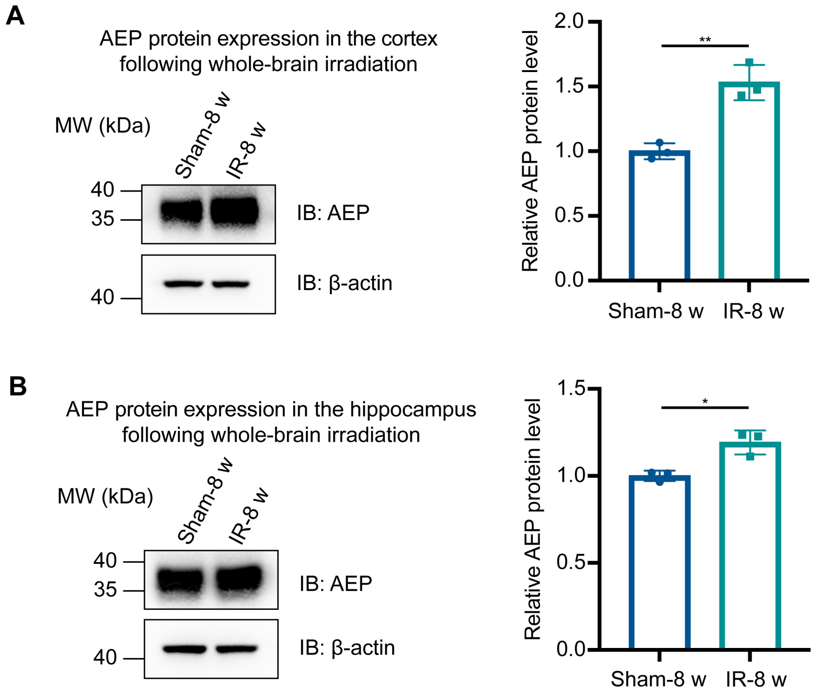

Figure S1. AEP protein expression was elevated 8 weeks following whole-brain irradiation, related to Figure 1

(A and B) Relative protein levels of AEP (left panel) and the quantification (right panel) in the cortex (A) and hippocampus (B) 8 weeks following whole-brain irradiation (n = 3 per group). AEP = asparagine endopeptidase; IB = immunoblotting; IR = irradiation. Data are represented as mean ± SD. Student's *t* tests were used in (A and B). * $P < 0.05$, ** $P < 0.01$.

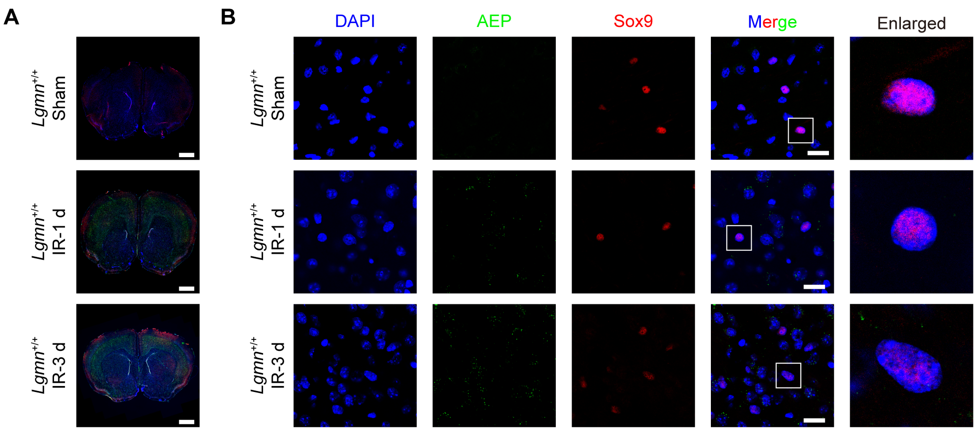

Figure S2. AEP expression was not elevated in astrocytes in the cortex following whole-brain irradiation, related to Figure 1

(A and B) Whole-brain section images (A, scale bar = 1 mm) and localized cortical images (B, scale bar = 20 μm) of immunofluorescence analysis of AEP and Sox9 following whole-brain irradiation. AEP = asparagine endopeptidase; LGMN = legumain; IR = irradiation.

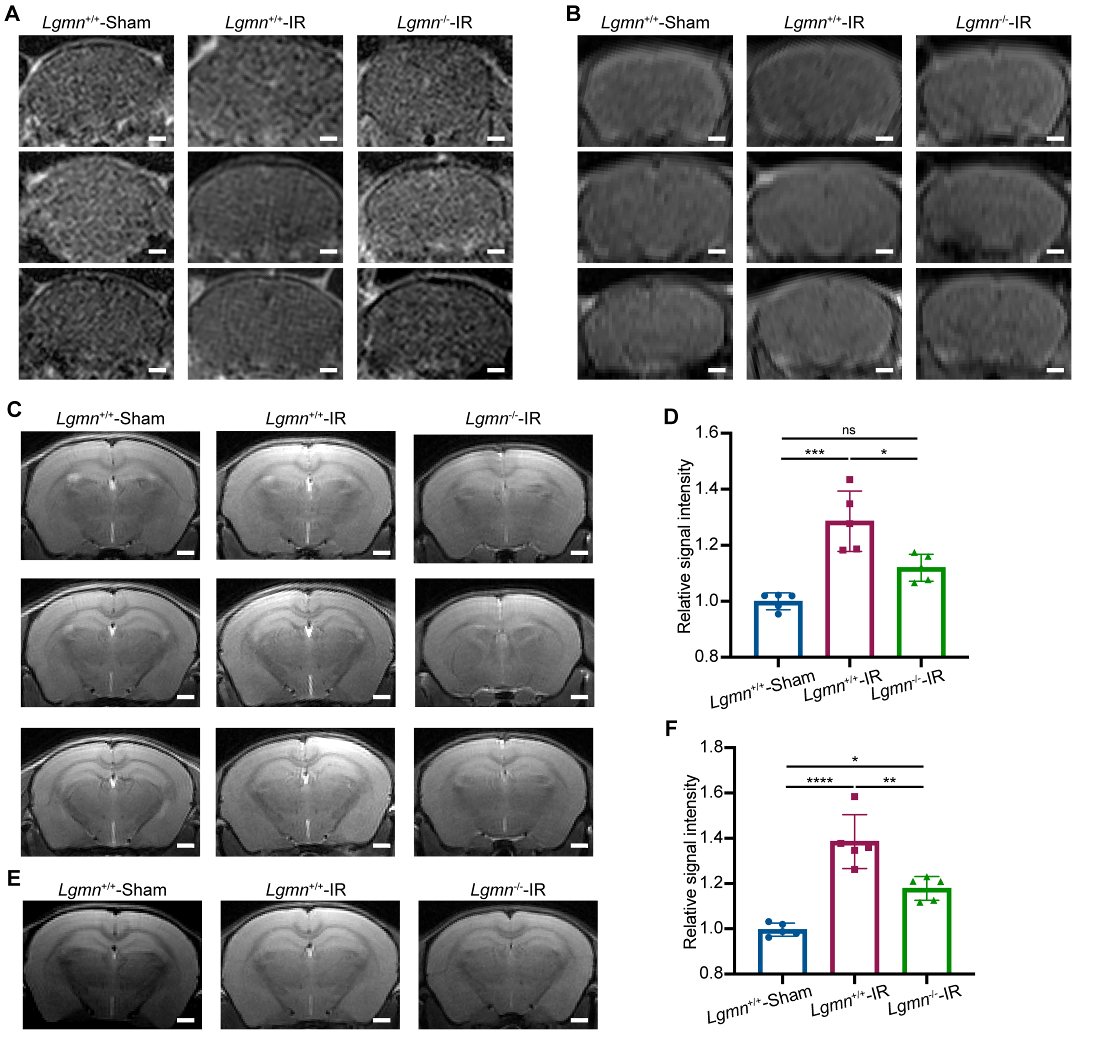

Figure S3. Radiation-induced brain injury was lessened by *Lgmn* knockout, related to Figure 3

(A and B) T1-weight MRI scan with contrast of the brain of *Lgmn*$^{+/+}$-Sham, *Lgmn*$^{+/+}$-IR and *Lgmn*$^{-/-}$-IR mice at 1 week (A) and 4 weeks (B) following whole-brain irradiation (scale bar = 1 mm, n = 5 per group).

(C and D) T1-weight MRI scan with contrast of the brain of *Lgmn*$^{+/+}$-Sham, *Lgmn*$^{+/+}$-IR and *Lgmn*$^{-/-}$-IR mice (C) and the quantification of MRI signal intensity of the cortex (D) at 6 weeks following whole-brain irradiation (scale bar = 1 mm, n = 5 per group; F value of genetic type = 13.94; F value of irradiation = 41.36).

(E and F) T1-weight MRI scan with contrast of the brain of *Lgmn*$^{+/+}$-Sham, *Lgmn*$^{+/+}$-IR and *Lgmn*$^{-/-}$-IR mice (E) and the quantification of MRI signal intensity of the cortex (F) at 8 weeks following whole-brain irradiation (scale bar = 1 mm, n = 5 per group; F value of genetic type = 18.15; F value

of irradiation = 64.00).

LGMN = legumain; IR = irradiation. Data are represented as mean ± SD. Two-way ANOVA tests were used in (D and F). * $P < 0.05$, ** $P < 0.01$, *** $P < 0.001$, **** $P < 0.0001$, ns = not significant.

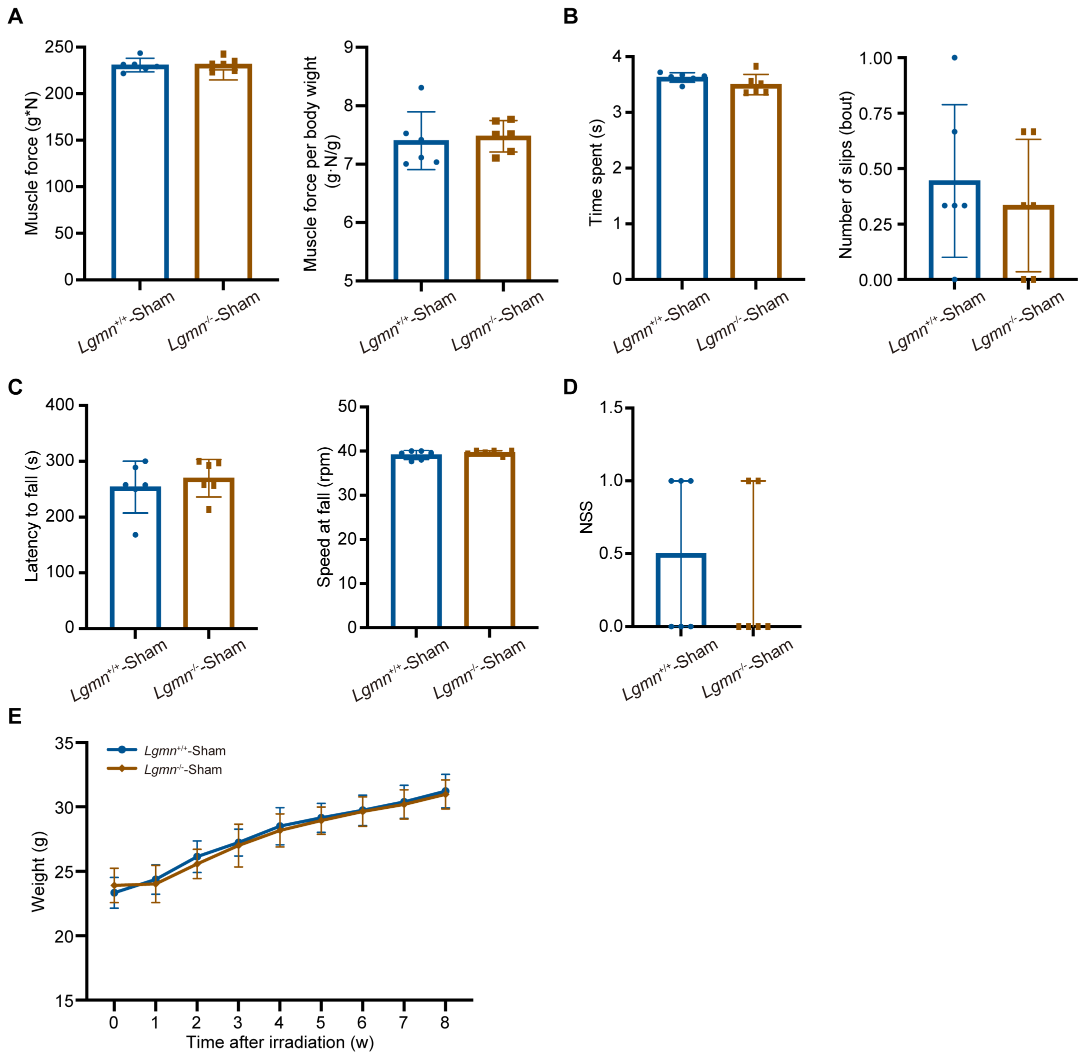

Figure S4. The neurological behavior was not different after *Lgmn* knockout in the sham groups, related to Figure 3

(A) Muscle force tests of *Lgmn*$^{+/+}$-Sham and *Lgmn*$^{-/-}$-Sham mice (n = 6 per group).

(B) Balance beam tests of *Lgmn*$^{+/+}$-Sham and *Lgmn*$^{-/-}$-Sham mice (n = 6 per group).

(C) Rotarod tests of *Lgmn*$^{+/+}$-Sham and *Lgmn*$^{-/-}$-Sham mice (n = 6 per group).

(D) Neurological severity score tests of *Lgmn*$^{+/+}$-Sham and *Lgmn*$^{-/-}$-Sham mice (n = 6 per group).

(E) Body weight of *Lgmn*$^{+/+}$-Sham and *Lgmn*$^{-/-}$-Sham mice (n = 6 per group).

LGMN = legumain; NSS = neurological severity score. Data are represented as median ± IQR in (D), whereas the other data are represented as mean ± SD. Student's *t* tests were used in (A-C and E), and Mann-Whitney U-test was used in (D).

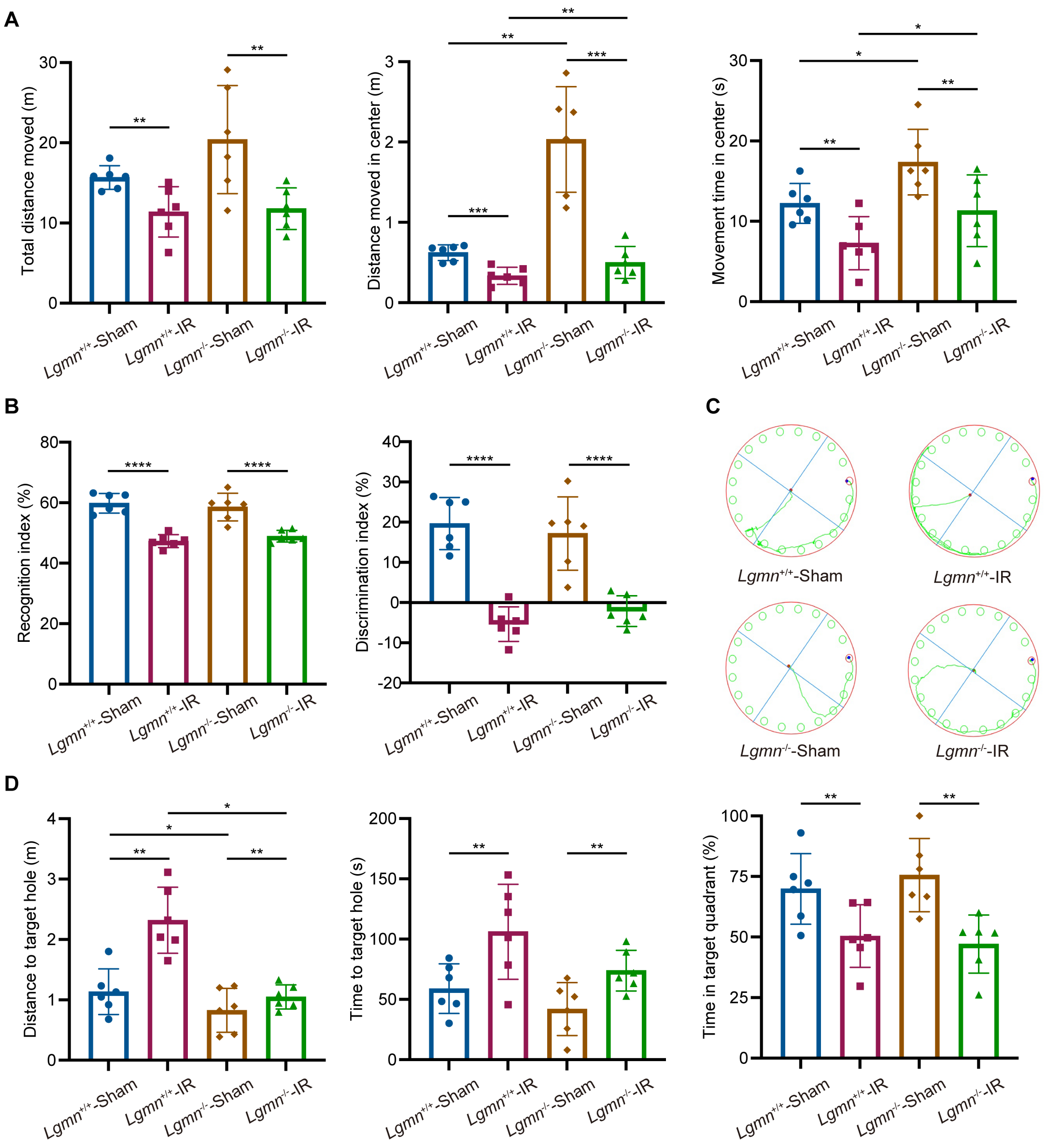

Figure S5. The hippocampal neurological impairment following whole-brain irradiation was partially reduced by *Lgmn* knockout, related to Figure 3

(A) Open field tests of *Lgmn*$^{+/+}$-Sham, *Lgmn*$^{+/+}$-IR, *Lgmn*$^{-/-}$-Sham and *Lgmn*$^{-/-}$-IR mice (n =6 per group). The left panel is the total distance moved in the whole field, the middle panel is the distance moved in the center, and the right panel is the time spent in the center (F value of genetic type = 2.38, 16.36 and 9.81 in the left, middle and right panel respectively; F value of irradiation = 15.01, 21.89 and 14.09 in the left, middle and right panel respectively).

(B) Novel object recognition tests of *Lgmn*$^{+/+}$-Sham, *Lgmn*$^{+/+}$-IR, *Lgmn*$^{-/-}$-Sham and *Lgmn*$^{-/-}$-IR mice (n = 6 per group). The left panel is the percentage of the time spent exploring the novel object divided by the time spent exploring both objects, and the right panel is the percentage of the time

spent exploring the novel object over the familiar object divided by the total exploration time (F value of genetic type = 0.02 and 0.02 in the left and right panel respectively; F value of irradiation = 73.53 and 73.53 in the left and right panel respectively).

(C and D) The representative tracks (C) and the quantification (D) of *Lgmn*$^{+/+}$-Sham, *Lgmn*$^{+/+}$-IR, *Lgmn*$^{-/-}$-Sham and *Lgmn*$^{-/-}$-IR mice in Barnes maze test (n = 6 per group). In (D), the left panel is the distance moved to the target hole, the middle panel is the time spent to the target hole, and the right panel is the percentage of time spent in the target quadrant (F value of genetic type = 9.81, 5.41 and 0.05 in the left, middle and right panel respectively; F value of irradiation = 14.09, 14.02 and 18.66 in the left, middle and right panel respectively).

LGMN = legumain; IR = irradiation. Data are represented as mean ± SD. Two-way ANOVA tests were used in (A, B and D). * $P < 0.05$, ** $P < 0.01$, *** $P < 0.001$, **** $P < 0.0001$.

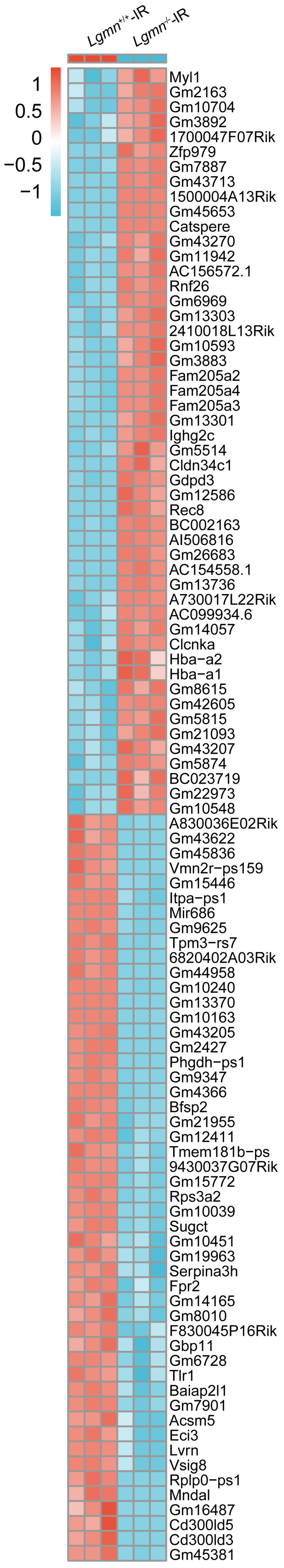

Figure S6. Heatmap of the bulk RNA sequencing data, related to Figure 5

The Heatmap represents the bulk RNA sequencing data of *Lgmn*$^{+/+}$-IR and *Lgmn*$^{-/-}$-IR mice cortex 3 days following whole-brain irradiation showing the top 50 significantly upregulated and downregulated genes.

LGMN = legumain.

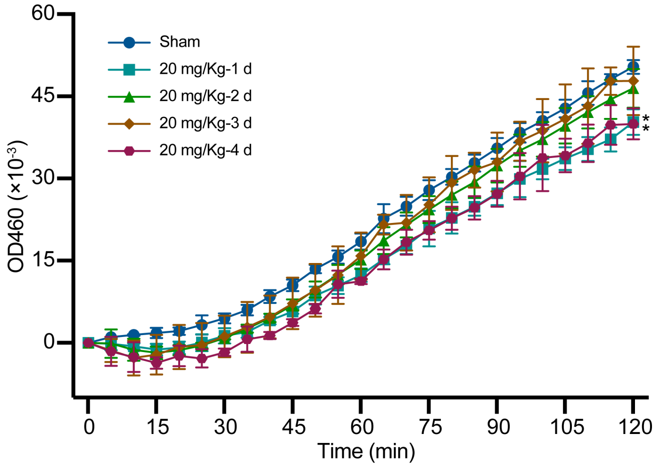

Figure S7. Enzymatic activity assay of AEP, related to Figure 7

AEP's enzymatic activity in the cortex of WT mice with or without intraperitoneal injection of 20 mg/kg esomeprazole (n = 3 per group; F value = 4.882).

AEP = asparagine endopeptidase. Data are represented as mean ± SD. One-way ANOVA tests were used. * $P < 0.05$.

Table S1. Primer sequence for tail genomic DNA validation, related to STAR Methods

Primer	Sequenced
5-3 primer	TAGCCTCTGTATAGCAACAGTG
C3 primer	CGTTGATGTCGTCGGGCA
Neo1 primer	CTCTATGGCTTCTGAGGCGGAAAG

Table S2. Primer sequence for RT-qPCR analysis, related to STAR Methods

Gene Symbol	Forward primer 5'→3'	Reverse primer 5'→3'
Lgmn	TGGACGATCCCGAGGATGG	GTGGATGATCTGGTAGGCGT
Gapdh	AGGTCGGTGTGAACGGATTTG	TGTAGACCATGTAGTTGAGGTCA
H2-T23	ACAGTCCCGACCCAGAGTAG	CCACGTAGCCGACAATGATGA
Hspa1b	GAGATCGACTCTCTGTTCGAGG	GCCCGTTGAAGAAGTCCTG
Raet1e	CCTCTGAACGATTTGTGCCAGG	GCCCTGGCTTTGCGGATAAATC
H2-D1	TCGGCTACTACAACCAGAGC	TAATGCTCTGCAGCACCACTCT
Gm7030	CCCTGACCTGGCAGTTGAAT	GTGTATCTAGGCTCCTCCCCA
Ide	CAGAAGGACCTCAAGAATGGGT	GCCTCGTGGTCTCTCTTTATCT
Il-1β	GCAACTGTTCCTGAACTCAACT	ATCTTTTGGGGTCCGTCAACT
Tnf-α	CCCTCACACTCAGATCATCTTCT	GCTACGACGTGGGCTACAG
Il-6	TAGTCCTTCCTACCCCAATTTCC	TTGGTCCTTAGCCACTCCTTC

Table S3. Neurological severity score test, related to STAR Methods

Task	Score
Presence of mono- or hemiparesis	1
Inability to walk on a 3-cm-wide beam	1
Inability to walk on a 2-cm-wide beam	1
Inability to walk on a 1-cm-wide beam	1
Inability to balance on a 0.7-cm-wide beam	1
Inability to balance on a 0.5-cm-wide round stick	1
Failure to exit a 30-cm-diameter circle in 2 minutes	1
Inability to walk straight	1
Loss of startle behavior	1
Loss of seeking behavior	1
The maximum total score	10
